# Different Leaf Anatomical Responses to Water Deficit in Maize and Soybean

**DOI:** 10.3390/life13020290

**Published:** 2023-01-20

**Authors:** Noel Anthony Mano, Bethany Madore, Michael V. Mickelbart

**Affiliations:** 1 Department of Botany and Plant Pathology, Center for Plant Biology, Purdue University, West Lafayette, IN 47907, USA; 2 Department of Horticulture and Landscape Architecture, Purdue University, West Lafayette, IN 47907, USA

**Keywords:** plasticity, stomatal development, abiotic stress, *Zea mays*, *Glycine max*

## Abstract

The stomata on leaf surfaces control gas exchange and water loss, closing during dry periods to conserve water. The distribution and size of stomatal complexes is determined by epidermal cell differentiation and expansion during leaf growth. Regulation of these processes in response to water deficit may result in stomatal anatomical plasticity as part of the plant acclimation to drought. We quantified the leaf anatomical plasticity under water-deficit conditions in maize and soybean over two experiments. Both species produced smaller leaves in response to the water deficit, partly due to the reductions in the stomata and pavement cell size, although this response was greater in soybean, which also produced thicker leaves under severe stress, whereas the maize leaf thickness did not change. The stomata and pavement cells were smaller with the reduced water availability in both species, resulting in higher stomatal densities. Stomatal development (measured as stomatal index, SI) was suppressed in both species at the lowest water availability, but to a greater extent in maize than in soybean. The result of these responses is that in maize leaves, the stomatal area fraction (*f_gc_*) was consistently reduced in the plants grown under severe but not moderate water deficit, whereas the *f_gc_* did not decrease in the water-stressed soybean leaves. The water deficit resulted in the reduced expression of one of two (maize) or three (soybean) *SPEECHLESS* orthologs, and the expression patterns were correlated with SI. The vein density (VD) increased in both species in response to the water deficit, although the effect was greater in soybean. This study establishes a mechanism of stomatal development plasticity that can be applied to other species and genotypes to develop or investigate stomatal development plasticity.

## 1. Introduction

Maize (*Zea mays* L.) and soybean (*Glycine max* (L.) Merr.) are mono and eudicotyledonous species, respectively, that are widely grown for food, feedstock, and industrial uses that represent approximately 35% of the total planted crop area in the United States [1]. The U.S. Midwest, where these crops are primarily grown, is expected to experience an increase in the frequency and the intensity of drought events [2], with subsequent severe reductions in yield [3]. Abnormal dry periods during regular periods of sufficient rainfall to support the growth and yield of these crops are also expected to become longer in the U.S., resulting in the soil water availability changing drastically in week-to-month timescales [4].

Crop plants that exhibit phenotypic plasticity may be more resilient to water deficit conditions [5]. Genotypes of maize that exhibit water-deficit-induced anatomical plasticity via narrower root growth angle [6], smaller root metaxylem and smaller distance between leaf vascular bundles [7], or increased allocation to reproductive biomass [8] have higher yield stabilities under water limitation compared to genotypes in which these traits are non-plastic. Soybean genotypes in which anatomical plasticity resulted in an increased root metaxylem number [9], thicker leaves, or faster transitions to the reproductive stage [10] were also better able to maintain yield stability under drought.

The plasticity in stomatal anatomy may improve resilience under water deficit conditions, as plants with lower stomatal density (SD, the number of stomata per unit epidermal area) often have lower rates of stomatal conductance [11,12]. Theoretical [13] and transgenic work [14,15,16] suggest that stomatal conductance can be reduced via a reduction in SD to some degree to enhance water-use efficiency and drought tolerance without adversely affecting photosynthesis, plant growth, or yield.

The stomatal size (SS) and density determine the stomatal area fraction (*f_gc_*), the percentage of epidermis occupied by stomata, which in turn is often correlated with leaf conductance [17,18]. Maize leaves typically produce smaller stomata at an increased density in response to water deficit (Supplementary Figure S1A,B). In soybean, smaller stomata have been reported in response to water deficit (Supplementary Figure S2A), but changes in the SD in response to water deficit are variable (Supplementary Figure S2B). The *f_gc_* was reported or could be calculated in very few maize or soybean studies. In most cases, the *f_gc_* did not change in response to water deficit, but it was reduced in specific genotypes, typically in response to a severe, not moderate, stress (Supplementary Figures S1 and S2). Although there are several reports characterizing epidermal traits in response to water deficit in these two species, few report the *f_gc_* on both leaf surfaces, and fewer still report stomatal development as estimated by stomatal index (SI) (Supplementary Figures S1 and S2). To date, there are no reports of comprehensive epidermal plasticity in either of these species.

The plasticity in the *f_gc_* cannot be assumed from a change in the SD or SS independently because, under water deficit conditions, the reduced turgor-driven cell expansion may result in changes in cell size and/or distribution that are independent of altered stomatal development. The reduced stomatal size with a concomitant increase in the SD typically results in no *f_gc_* plasticity, and this negative scaling relationship between SD and SS has been well established [19,20]. However, water-deficit-induced reductions in stomatal development do occur [21,22], and may contribute to a reduction in the *f_gc_*. In maize, water deficit often does not result in changes to SI (Supplementary Figure S1C) but increases in the SI have been reported [6,23]. In soybean leaves, Tripathi et al. [24] observed a reduction in the SI and SD on the abaxial surface. However, none of these studies included measurements of stomatal dimensions, so the potential effect of altered stomatal development on the *f_gc_* was not established.

The plasticity of stomatal development may at least partially be the result of a reduced expression of the transcription factor *SPEECHLESS* (*SPCH*), which facilitates the initial entry of stem cells into the stomatal development pathway in eudicots [25] and monocots [26]. In Arabidopsis, stomatal development is inhibited by water deficit [21,22] via transcriptional repression of *SPCH* expression [27,28] and post-translational modification of the SPCH protein [21], whereas expression of two *SPCH* orthologues was downregulated several hours after a detachment and dehydration treatment of soybean leaves [24]. *SPCH* expression may also play a role in the adaptation to water availability. In response to drought, *SPCH* expression was downregulated in one of two black poplar genotypes [29] and the timing of expression of *SPCH* differs to coordinate distinct stomatal development stages in terrestrial and amphibious members of the genus *Callitriche* [30]. These reports together point to suppressed *SPCH* expression as a strong candidate mechanism to facilitate stomatal development inhibition in water-stressed plants. However, to date, there are no reports of *SPCH* expression in maize or soybean in response to rootzone water deficit [31].

In addition to the lack of complete epidermal characterization in previous studies, many published studies consist of rapidly imposed water deficits, as opposed to a maintained water deficit through the leaf development period, which is more similar to field conditions. Therefore, the leaf plasticity response to water deficit and its underlying mechanisms in maize and soybean remain unknown. We aimed to characterize the leaf anatomical response to a controlled water deficit in temperate genotypes of maize (B73 × Mo17) and soybean (IA3023), as agriculturally important species with grass and eudicot stomatal anatomy, respectively. We quantified the arrest of stomatal development under the water deficit, and then examined the importance of this inhibition to the *f_gc_* response. We further hypothesized that reduced *SPCH* expression leads to stomatal development inhibition and thus decreased SI. This study shows that minimal reduction in cell size and stomatal development inhibition in water-stressed maize results in a lower stomatal area fraction, whereas in the water-stressed soybean, the reduction in cell size and minimal inhibition of stomatal development conserves the stomatal area fraction. This study also provides novel direct evidence for the regulation of stomatal development via the reduced *SPCH* expression in maize and soybean.

## 2. Materials and Methods

### 2.1. Plant Material, Growth Conditions, and Imposition of Water Deficit

A greenhouse experiment was conducted with soybean variety IA3023 and the maize hybrid B73 × Mo17 in 2018, and then repeated in 2019. IA3023 was chosen as a high-yielding elite line well-adapted to the local climate [32], whereas B73 × Mo17 is a well-established hybrid line [33] derived from well-characterized inbred parents [34]. The temperature and relative humidity were recorded by HOBO loggers (Onset Computer Corporation, Bourne, MA, USA) on the greenhouse benches, and the photosynthetically active radiation was recorded by a weather station outside the greenhouse (Supplementary Figure S3). Supplemental lighting was supplied at 160 μmol m^−2^·s^−1^ as needed to maintain a minimum photoperiod of 12 h. Acidified water was supplemented with water-soluble fertilizer (ICL Specialty Fertilizers, Dublin, OH, USA) to provide the following (in mg L^−1^): 150 N, 9.8 P, 119 K, 12 Mg, 21 S, 1.5 Fe, 0.4 Mn and Zn, 0.2 Cu and B, and 0.1 Mo. Nitrate and ammoniacal sources of nitrogen were provided as 61 and 39% total N, respectively. The irrigation water was supplemented with 93% sulfuric acid (Brenntag, Reading, PA, USA) at 0.08 mL L^−1^ to reduce the alkalinity to 100 mg L^−1^ and pH to a range of 5.8 to 6.2.

Plants were grown in a soilless media of three parts by volume Berger BM8 soilless media (Berger, Saint Modeste, QC, Canada) to one part by volume Turface Athletics MVP calcined clay (PROFILE Products LLC, Buffalo Grove, IL, USA) in 3.8 L pots. The media water content (MWC) was maintained at one of four levels: 100, 75, 50, or 30%. The MWC was calculated as

MWC = [(MW − MDW)/(MSW − MDW)] × 100(1)

where MW is the weight of the system at a given time, MDW is the dry weight of the media, and MSW is the weight of the system when thoroughly saturated with water. The stress was initiated at the V1 or T3/T4 stage for maize and soybean, respectively, in experiment 1, and at the V1 and unifoliate leaf stage in experiment 2. Water was withheld from treatment containers until the target MWC was achieved, at which point the containers were weighed and irrigated every two days to maintain the treatment MWC levels.

### 2.2. Leaf Sampling and Data Collection

After the target MWC was reached, the emerged leaves were tagged to ensure that anatomical data was collected from leaves that developed exclusively under the desired treatment conditions. In experiment 1, the sampled leaves were the fourth and fifth soybean trifoliate leaves (T4 and T5) and the seventh maize leaf. In the second experiment, the third soybean trifoliate leaf (T3) and eighth maize leaf were collected. The treatment MWC was maintained for 20 to 30 days, which was the time required for post-stress leaves to emerge and develop to full expansion. The full expansion was confirmed in the soybean by photographing the leaves and confirming no further increase in the leaf area over a period of several days. The maize leaves that had developed a prominent collar were considered fully expanded.

### 2.3. Plant Growth and Water Relations Traits

In the second experiment, 14 maize leaves (of the 28 total experimental replicates) were removed and photographed to obtain the leaf area using ImageJ software. Length (L) and width (W) were quantified in all leaves and the equation

LA (cm^2^) = 0.7597(L × W) − 6.6523 (2)

was used to derive the maize leaf area from the maize leaf length and width in both experiments. The soybean leaves were photographed, and the leaflets were traced to obtain the leaf area.

An 8-cm section from the middle of each maize leaf or half of a soybean leaflet was weighed to obtain the fresh weight (FW), then floated on water in a 50 mL tube and kept under non-saturating light for 6 to 8 h, at which point the leaf pieces were blotted dry and weighed to obtain the turgid weight (TW), then dried at 65 °C for at least 48 h to obtain the dry weight (DW). The relative water content (RWC, %) was calculated as

RWC (%) = [(FW − DW)/(TW − DW)] × 100.(3)

The portion of the leaf used for the RWC quantification was scanned when the turgid weight was obtained to determine the area (LA), from which the specific leaf weight (SLW, mg cm^−2^) was calculated as DW/LA.

To measure the osmotic potential, the leaf tissue was cut and placed in a dolphin tube with a Costar Spin-X 8163 insert (Cole-Parmer, Vernon Hills, IL, USA) and then immediately stored in liquid nitrogen for transport back to the laboratory. Leaf samples for osmotic potential were kept in a −80 °C freezer before being thawed in their sealed tubes and centrifuged for 5 min at 120 RPM × 100 to extract sap. The osmolality was quantified from 10 μL of sap using a vapor pressure osmometer (VAPRO 5520; Wescor Inc., Logan, UT, USA). The osmolality was converted to osmotic potential (Ψ_s_, MPa) as

Ψ_s_ = −CRT, (4)

where C is osmolality, R is the gas constant, and T is temperature. From the osmotic potential and RWC, the osmotic potential at full turgor (Ψ_100_) was calculated as

Ψ_100_ = Ψ_s_ × (RWC/100). (5)

Osmotic adjustment (OA) was assumed to occur if Ψ_100_ was significantly different from the control plants at a given treatment level.

### 2.4. Anatomical Traits

In both experiments, the stomatal anatomy was quantified from the lamina half-way between the tip and the collar of the maize leaves, and the soybean leaflet lamina half-way between the base and the tip and between the midrib and edge of the leaflet. In experiment 1, epidermal impressions of the adaxial and abaxial surfaces were made using cyanoacrylate (Duro Super Glue; Henkel, Düsseldorf, Germany) on glass slides. In the second experiment, leaf segments were placed in envelopes and stored at −20 °C until further processing. Leaves were then cleared in a solution of 9:1 ethanol-to-acetic acid in a 60 °C incubator. After the initial clearing in the ethanol-to-acetic acid, maize and soybean leaf tissue were cleared as in McAdam et al. [35]. Soybean leaves were cleared with bleach in a vacuum desiccator, then stained with 0.015% toluidine blue. To quantify the stomatal anatomy from both epidermal impressions and cleared leaves (Supplementary Figure S4), four images (100× and 200× for maize and soybean, representing areas of 0.48 and 0.13 mm^2^, respectively) were taken from each surface of each leaf with a DCM 900 microscope CMOS Camera (Oplenic Optronics, Hangzhou, China).

The number of stomata and pavement cells per unit area (SD and PD, respectively) was counted using ImageJ software. Only whole stomata and pavement cells and partial cells bordering the top and right sides of each image were counted. The stomatal index (SI) was calculated as

SI (%) = [Ns/(Ns + Np)] × 100,(6)

Where Ns represents the number of stomata, and Np represents the number of pavement cells. The soybean SS was calculated using the formula for an ellipse,

SS (μm^2^) = π × (SL/2) × (SW/2), (7)

where SL and SW represent the stomatal length and width, respectively. In maize, the SS was calculated using the formula for a rectangle, SL × SW. The stomatal area fraction (*f_gc_*) was calculated as in Sack et al. [18]:

*f_gc_* = (SS × SD)/100. (8)

The pavement cell size was estimated by dividing the number of pavement cells counted in a region and dividing by the size of the respective visual field for each species, after using the *f_gc_* to subtract the total area of the stomata occupying the field.

In the second experiment, the same cleared tissue used to quantify stomatal traits was imaged at 40× magnification to visualize venation. Veins were traced in ImageJ and the total vein length measured per image was divided by the area of the field of view (2.97 mm^2^) to obtain the vein density (mm of veins per mm^−2^ area).

### 2.5. Gene Expression Analysis

In the second experiment, the leaf tissue from an additional three plants per treatment × species was collected seven to ten days after the target MWC was reached, as leaves equivalent to those used for anatomical and physiological measurements were emerging. The third soybean trifoliate leaf was collected when the unfolded leaves were ca. 2 cm in length. In maize, the eighth leaf was harvested from within the leaf sheath when the sixth leaf had emerged but not yet collared. This eighth leaf segment was partitioned into four zones: 1 cm of tissue from the leaf base upwards, the next cm above this zone (1–2 cm above the leaf base), then a 4 cm segment from 2–6 cm above the leaf base, and finally a 4–8 cm segment beginning at 6 cm above the leaf base, depending on the length of the leaf that remained (Supplementary Figure S5). In maize, gene expression was quantified from the first and fourth segments to reliably use leaf tissue in the cell proliferation/differentiation stage and fully expanded tissue [36]. The middle two stages were not examined for gene expression because it is likely that the two zones are variable across water deficit treatments [37]. Therefore, we would confound the effects of water deficit with those of stage differences. Moreover, Raissig et al. [26] showed that the partially differentiated stomatal complexes observed in the middle leaf zones no longer had detectable *SPCH* expression. All tissue was frozen in liquid nitrogen immediately after segmenting. In soybean, an unfolded T3 trifoliate leaf, which also has primarily differentiating cells [38], was used to quantify *SPCH* expression. We quantified the expression of two and three homologues of *SPCH* in maize and soybean leaves, respectively, based on the identification of bHLH stomatal development gene families, including *SPCH*, by Ran et al. [39], with two important caveats. First, GRMZM2G064638 is now considered a homologue of *MUTE* [40]. Accordingly, we have named GRMZM2G045109 (Zm00001d046096) and GRMZM2G085751 (Zm00001d053391) as *ZmSPCH1* and *ZmSPCH2*. Secondly, Glyma14g31385 (now Glyma14g160700; *GmSPCH4*) showed no detectable gene expression in any tissue of young soybean seedlings [41], and so was not investigated here.

The leaf tissue was ground in liquid nitrogen before extracting the RNA using the RNeasy PowerPlant Kit (Qiagen, Germantown, MD, USA). Residual DNA was removed from the extracted RNA using the Qiagen DNAse Max Kit. In total, 2 μg of cDNA was synthesized from the extracted RNA using the SuperScript III First-Strand Synthesis System (ThermoFisher Scientific, Waltham, MA, USA). All procedures were performed as directed in the manufacturer protocols.

The synthesized cDNA was used in a 1:40 dilution with autoclaved water for use in the qPCR. The qPCR was performed using a Luna Universal qPCR Master Mix with SYBR Green chemistry (New England Biolabs, Ipswich, MA, USA), in a StepOne Plus thermocycler (Applied Biosystems, Foster City, CA, USA). Three biological replicates per treatment and three technical replicates per biological replicate were used for a given *SPCH* orthologue or control gene. *ZmTUBULIN1* and *ACTIN11* were used as control genes in the maize and soybean, respectively. Ct values for each gene were compared using the double-delta method [42] to obtain the relative expression of each *SPCH* orthologue in the different treatments.

### 2.6. Data Analysis and Experimental Statistics

In both experiments, maize and soybean plants were grown separately on adjacent greenhouse benches to avoid shading. To analyze the response of plant and epidermal traits, experimental data were analyzed across categorical treatment groups within each species. The first experiment was a completely randomized design with five soybean plants and four maize plants per treatment. The second experiment was a randomized complete block design, with eight plants per treatment per species. In the first experiment, a one-way ANOVA with post-hoc Tukey or Games-Howell test was used, depending on the equivalence of variance among treatment groups (Supplementary Table S1). In this experiment, the soybean SD was significantly different between T4 and T5, so anatomical data were analyzed separately within each leaf group. In the second experiment, a mixed model was used wherein block and treatment were treated as random and fixed effects, respectively. As experiments were conducted in different years and different leaf numbers were sampled in each experiment, the data from experiments one and two were analyzed and are presented separately. A post-hoc test with a Bonferroni correction was used to identify the difference between fixed-effect (water-deficit treatment) groups (Supplementary Table S2). Where necessary, Box-Cox or Johnson transformations were used to normalize the data. All statistical analyses were performed in JMP Pro 14 [43]. A list of gene accession numbers and associated primer sequences is given in Supplementary Table S3.

## 3. Results

### 3.1. Effect of Water Deficit on Plant Water Status and Growth

The average daytime greenhouse temperature and vapor pressure deficit (VPD) was ~2 °C and 0.2 kPa higher, respectively, over the duration of the second experiment compared to the first experiment (Supplementary Figure S3). Possibly as a result, media in the second experiment reached a lower MWC prior to irrigating in some cases, relative to the media in the first experiment (Supplementary Figures S6 and S7). This difference was especially pronounced for soybean plants.

**Figure 1:**
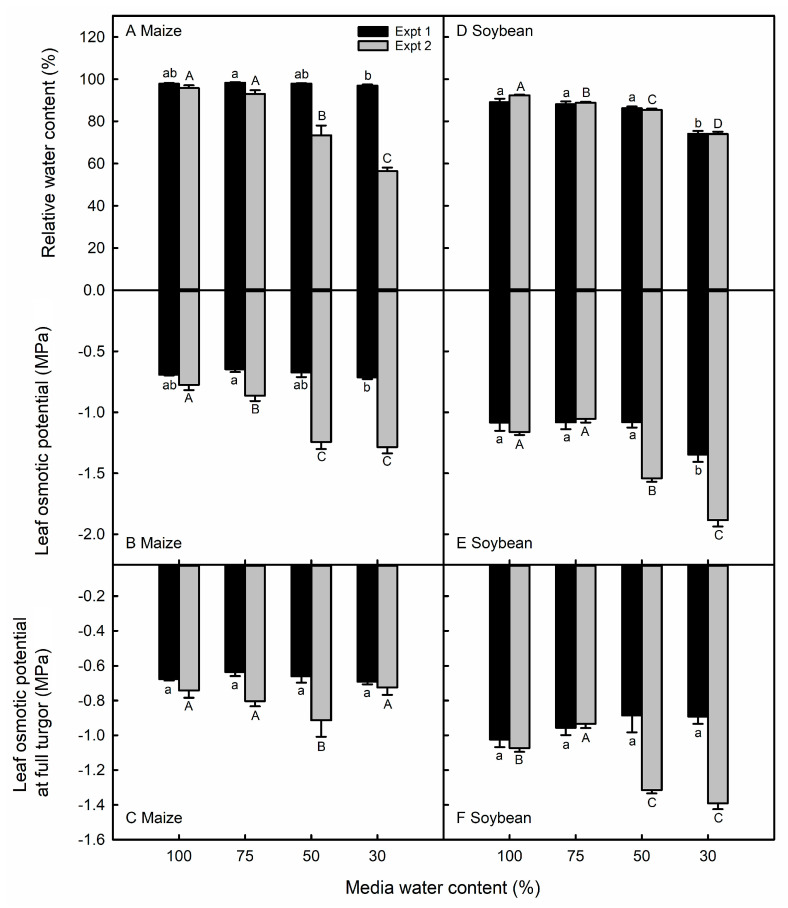
Relative water content (**A**,**D**), osmotic potential (**B**,**E**), and osmotic potential at full turgor (Ψ_100_; (**C**,**F**)) in maize and soybean leaves in response to different media water content treatments in the first (black bars) and second (grey bars) experiments. Data are shown as means ± standard errors for *n* = 4 maize plants and 5 soybean plants in the first experiment and *n* = 8 in the second experiment. Different letters above the columns indicate statistically significant differences between treatments within experiment 1 (lowercase) and experiment 2 (uppercase) leaf surfaces, as tested with a one-way ANOVA and mixed linear model and post-hoc test with Bonferroni correction, respectively.

The relative water content was lower in the moderately (50% MWC) and severely stressed (30% MWC) maize than in the control plants in the second, but not the first, experiment (Figure 1A). Similarly, the leaf osmotic potential (Ψ_s_) decreased with MWC in 50 and 30% MWC maize leaves of the second, but not the first, experiment (Figure 1B). In both experiments, there was no evidence of OA in maize in response to the water deficit stress, except in 50% MWC plants in the second experiment (Figure 1C).

In the first experiment, the soybean RWC was lower only in the 30% MWC plants, whereas the leaf RWC was reduced in all water-deficit stressed plants in the second experiment (Figure 1D). In both experiments, the soybean Ψ_s_ decreased only under low MWC conditions (Figure 1E). Soybean leaves in the first experiment did not osmotically adjust as the MWC decreased, but OA was observed in the 50% and 30% MWC treatments of the second experiment (Figure 1F).

**Figure 2:**
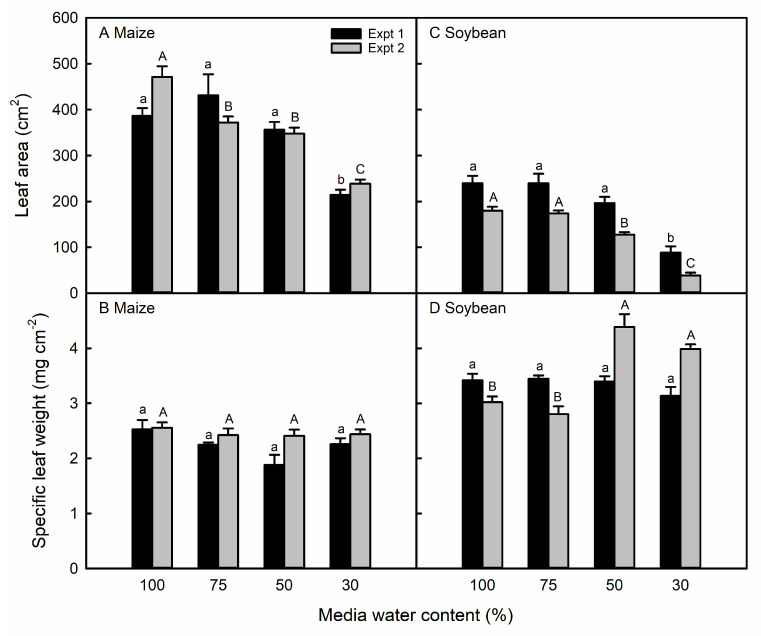
Leaf area (**A**,**C**) and specific leaf weight (**B**,**D**) in maize and soybean leaves in response to different media water content treatments in the first (black bars) and second (gray bars) experiments. Data are shown as means ± standard errors for *n* = 4 maize plants and 5 soybean plants in the first experiment and *n* = 8 in the second experiment. Different letters above the columns indicate statistically significant differences between treatments within experiment 1 (lowercase) and experiment 2 (uppercase) leaf surfaces, as tested with a one-way ANOVA and mixed linear model and post-hoc test with Bonferroni correction, respectively.

In the first experiment, only the 30% MWC treatment resulted in smaller maize leaves relative to the control plants, whereas in the second experiment, water-stressed maize leaves were smaller at all water deficit levels (Figure 2A). Maize leaf thickness, measured via SLW, was not affected by any degree of the water deficit in either experiment (Figure 2B). Soybean leaf size only decreased in the 30% MWC treatment of the first experiment, but the leaf size decreased relative to that of the control plants in the 50% and 30% MWC treatments in the second experiment (Figure 2C). All levels of water deficit resulted in no change in the soybean SLW in the first experiment, but in the leaves subjected to the 50% and 30% MWC treatments of the second experiment, the SLW was higher relative to that of well-watered plants (Figure 2D).

### 3.2. Stomatal Aantomical Plasticity in Response to Water Deficit

**Figure 3:**
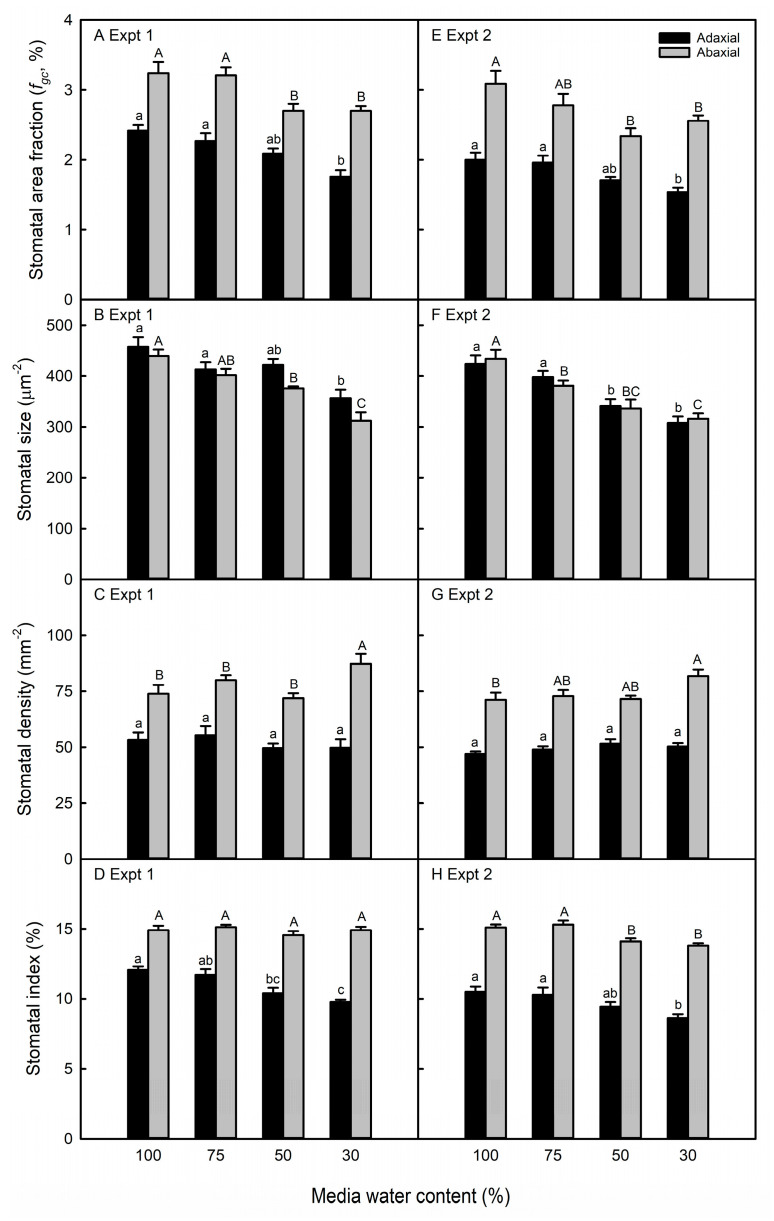
Stomatal area fraction (**A**,**E**), stomatal size (**B**,**F**), stomatal density (**C**,**G**), and stomatal index (**D**,**H**) of maize leaves in response to different media water content treatments in the first (**A**–**D**) and second (**E**–**H**) experiments. Data are shown as means ± standard errors for *n* = 4 in the first experiment and *n* = 8 in the second experiment. Different letters above the columns indicate statistically significant differences between treatments within adaxial (lowercase) and abaxial (uppercase) leaf surfaces. In the first experiment, treatment groups were compared with a one-way ANOVA and post-hoc Tukey or Games-Howell test, depending on the equivalence of variance between treatment groups. In the second experiment, treatment groups were compared with a mixed linear model and post-hoc test with Bonferroni correction.

The maize abaxial *f_gc_* was reduced by an average of 15% in the two lowest MWC treatments relative to that of the control plants across both experiments (Figure 3A,E). On the adaxial surface, the *f_gc_* was reduced by 25% only in plants grown under 30% MWC. This was a result of the water-stressed maize leaves producing smaller stomata (Figure 3B,F) with little to no change in the SD across all treatments (Figure 3C,G). Maize pavement cells were only slightly smaller in water-stressed leaves, being 20% smaller in the second experiment only (Supplementary Figure S8A,B). With little reduction in the pavement cell size, stomatal spacing was maintained, which explains the small increase in the SD. Another consequence of the small changes in stomatal frequency was that the distribution of abaxial-to-adaxial stomata was unresponsive to any level of water deficit in maize (Supplementary Figure S8C,D). The abaxial SI was lower in maize plants grown at 50% and 30% MWC only in the second experiment, whereas the severe water deficit resulted in a decreased SI on the adaxial leaf surface in both experiments (Figure 3D,H). Overall, the mildest water deficit treatment (75% MWC) resulted in no stomatal plasticity.

**Figure 4:**
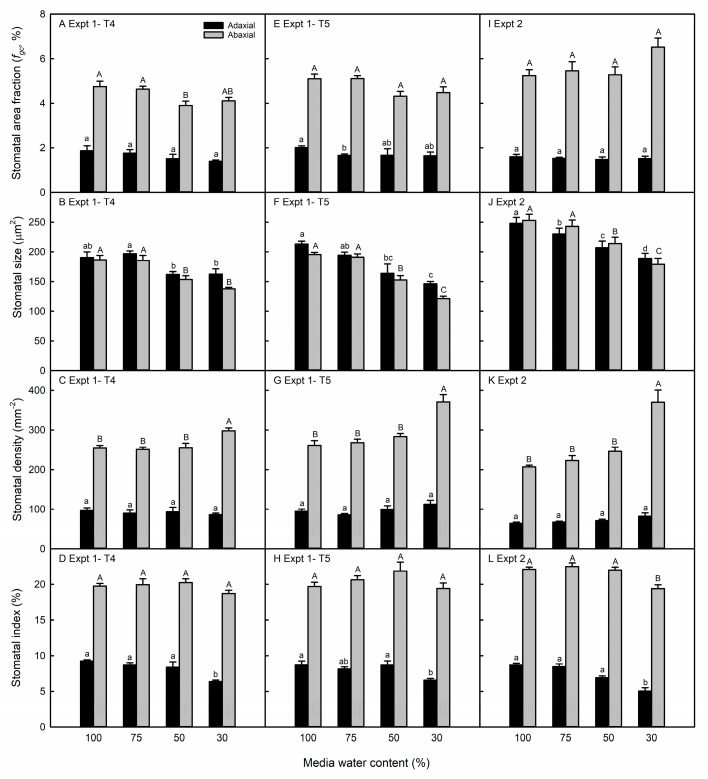
Stomatal area fraction (**A**,**E**,**I**), stomatal size (**B**,**F**,**J**), stomatal density (**C**,**G**,**K**), and stomatal index (**D**,**H**,**L**) of soybean leaves in response to different media water content treatments in the first (**A**–**H**) and second (**I**–**L**) experiments. In the first experiment, data are shown separately for the T4 (**A**–**D**) and T5 trifoliate leaves (**E**–**H**). Data are shown as means ± standard errors for *n* = 5 in the first experiment and *n* = 8 in the second experiment. Different letters above the columns indicate statistically significant differences between treatments within adaxial (lowercase) and abaxial (uppercase) leaf surfaces. In the first experiment, treatment groups were compared with a one-way ANOVA and post-hoc Tukey or Games-Howell test, depending on the equivalence of variance between treatment groups. In the second experiment, treatment groups were compared with a mixed linear model and post-hoc test with Bonferroni correction.

In the water-stressed soybean, the abaxial *f_gc_* was similar between the well-watered and water-stressed plants (Figure 4A,E,I). This was because while the water-stressed soybean stomata were smaller than those of the well-watered leaves (Figure 4B,F,J), unlike maize, the pavement cells were also smaller (Supplementary Figure S9A,B), resulting in a high abaxial SD in the leaves of the most severe water-deficit treatment (Figure 4C,G,K). There was no change in the SD on the adaxial surface, resulting in an increased abaxial-to-adaxial SD ratio in the 30% MWC soybean leaves (Supplementary Figure S9C,D). The soybean abaxial SI was reduced only at the most severe treatment level and only in the second experiment, whereas the abaxial SI decreased in the 30% MWC treatment in both experiments (Figure 4D,H,L).

**Figure 5:**
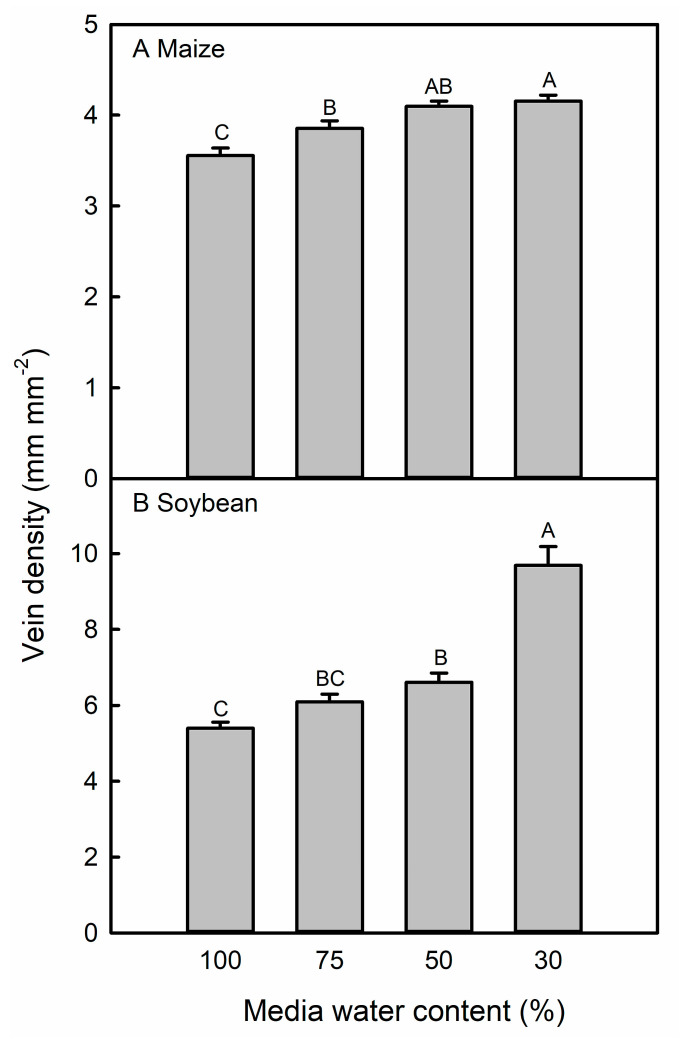
Vein density in maize (**A**) and soybean (**B**) leaves in response to different media water content treatments in the second experiment. Data are shown as means ± standard errors for *n* = 8. Different letters above the columns indicate statistically significant differences between treatments within each species. Treatment groups were compared with a mixed linear model and post-hoc test with Bonferroni correction.

In maize and soybean leaves, most levels of water deficit resulted in higher vein density (VD) (Figure 5), but the ratio of stomata to veins was largely unchanged in response to the MWC treatment (Supplementary Figure S10).

### 3.3. Gene Expression Changes Partially Explain Observed Changes in Stomatal Development

**Figure 6:**
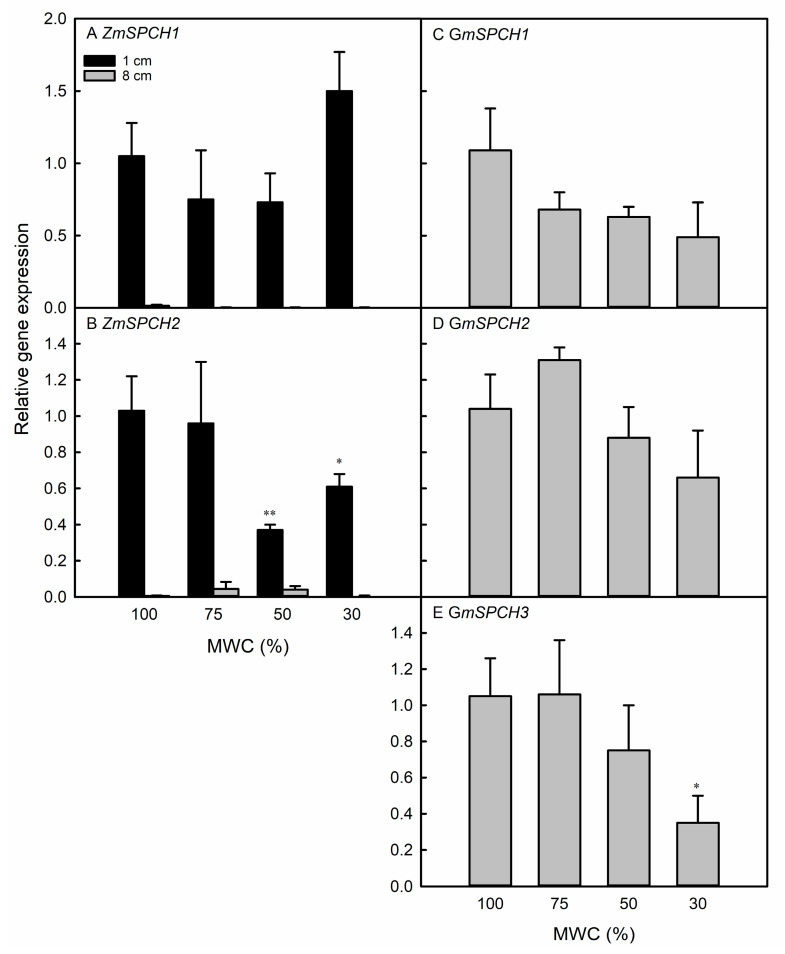
Expression of *SPCH* orthologs in maize (**A**,**B**) and soybean (**C**–**E**) leaves subjected to the indicated water deficit treatments, shown as a relative value to the 100% MWC control group. Data are presented as means ± standard error for three plants per species per treatment. In maize, data are shown from the leaf region of cell differentiation (1 cm) and region of mature leaf tissue (8 cm). Letters above columns indicate statistically significant differences between a treatment group against the well-watered control group, as tested with a mixed linear model. The * and ** indicate a statistically significant difference between a treatment group and the well-watered control.

We observed a change in the expression of distinct *SPEECHLESS* (*SPCH*) orthologs in both the maize and soybean leaves. In maize, *ZmSPCH2* expression was reduced in the region of developing (but not in more differentiated tissue) leaf tissue in the 50% and 30% MWC treatments, whereas *ZmSPCH1* expression was unresponsive to the water deficit (Figure 6A,B). Three orthologues of *SPCH* (*GmSPCH1*, *GmSPCH2*, and *GmSPCH3*) were examined in soybean leaves. Of these three, only *GmSPCH3* showed a significant reduction in expression in the 30% MWC treatment (Figure 6C–E).

## 4. Discussion

### 4.1. Maize and Soybean Exhibited Different Water-Relations Responses to Water Deficit

We imposed similar treatments of water deficit on the same maize and soybean genotypes in the two experiments (Supplementary Figure S6). However, the plants experienced higher levels of stress by the treatments imposed in the second experiment. For example, in the first experiment, we observed neither RWC response to decreasing the MWC in maize leaves (Figure 1A), nor OA in soybean leaves (Figure 1F), whereas these responses were observed in the second experiment. In both species, the leaf area decreased by a greater magnitude in the second experiment compared with the first (Figure 2A,C). Despite the similar MWC treatments imposed, plants in the second experiment experienced a higher average daytime temperature and VPD (27.4 °C and 1.55 kPa) as compared with the first experiment (25.1 °C and 1.31 kPa) (Supplementary Figure S3). As the treatment MWC was reached and harvested leaves were beginning to develop about ten days after the treatment initiation, the VPD was higher in the second experiment than at the comparable period in the first experiment (Supplementary Figure S3C,G). Additionally, the data collection from maize and soybean plants in the second experiment occurred during a period of increasing VPD, whereas the temperature and VPD decreased at the end of the first experiment (Supplementary Figure S3C,G).

The treatment application was similar in both experiments in that the water deficit was imposed prior to the emergence and through full expansion of the harvested leaves. Additionally, although the leaf numbers were different, they were developmentally similar in the two experiments, and in soybean, in different leaves in the same experiment (Figure 4A–H). The maize V7 and V8 leaves used for stomatal anatomy are both adult leaves [44,45], whereas the soybean T3–T5 leaves are all transitory between the juvenile andadult phases [46]. At the same time, the stomatal size and/or the area fraction were similar in the two experiments in maize and soybean, even though data were collected from epidermal impressions in the first experiment and the direct imaging of cleared tissue in the second experiment (Figure 3 and Figure 4). Therefore, we attribute the differences observed in the two experiments to be primarily the result of environmental, rather than developmental, variation or methodological differences.

Decreased Ψ_100_ occurred in severely stressed soybean leaves, indicating OA. OA has been reported in prior investigations of the drought-stressed soybean [47,48]. Sloane et al. [47] also attributed the improved drought tolerance in an introduced genotype to the greater capacity for OA. In the second experiment, soybean (but not maize) Ψ_100_ was correlated with the SD, SS, total cell number, and leaf area (Supplementary Table S4), supporting the idea that stomatal plasticity in soybean was comparatively limited because the greater OA was sufficient for drought tolerance. The OA that occurred in soybean may have helped to maintain the RWC under water stress, unlike in the maize plants in the second experiment (Figure 1). OA does occur in maize [49,50], including in the genotype used in our study [51,52]. OA may not have been observed in our study as we used a regular deficit irrigation, to which plants respond differently than they do when water is withheld [53,54], as occurred in these previous investigations.

### 4.2. Maize and Soybean Exhibited Different Anatomical Plastcity to Water Deficit

The few previously published experiments report a non-plastic response of the *f_gc_* to water deficit in maize [55,56,57]. In our study, we observed maize stomatal plasticity due to the stomatal development inhibition relative to the decrease in leaf and epidermal size. Under the most severe water deficit, maize leaves were 49% smaller than those of the control plants, whereas pavement cell size was unchanged in the first experiment and 25% smaller in the second experiment. As a result, the inhibited stomatal development (lower SI) was sufficient to prevent the stomatal density increase and weaken the negative correlation between the SD and SS (Supplementary Figure S11A). Therefore, the smaller stomata led to lower *f_gc_* in the water-stressed maize. Soybean water-stressed leaves were as much as 70% smaller than well-watered plants. Additionally, the soybean epidermal cell size decreased by 25% in the first experiment and up to 67% in the second experiment, so the ~15% decrease in the SI was not sufficient to prevent the large increase in the soybean SD. Therefore, our results suggest that soybean leaves of this genotype do not exhibit plasticity in the *f_gc_* because the reduction in SS, as a consequence of the water deficit, is largely counterbalanced by an increase in stomatal density (Supplementary Figure S11B).

Stomatal frequency or developmental plasticity usually occurs similarly on both leaf surfaces, such that the abaxial-to-adaxial stomatal ratio is not changed in water-stressed leaves relative to the control maize and soybean plants (Supplementary Figures S1 and S2). However, when the stomatal ratio did change, it increased in three soybean genotypes and decreased in two (Supplementary Figure S2). The increased abaxial-to-adaxial stomatal ratio, observed in soybean but not in maize, may also be an acclimation response to the water deficit. Amphistomatous plants have a higher stomatal conductance than do hypostomatous plants [58,59,60]. Additionally, as the ratio of the abaxial-to-adaxial stomata increases within the amphistomatous species, mesophyll and stomatal conductance decrease [60]. This can be explained by the incidence of light on the adaxial surface inducing stomatal opening and increasing evaporative demand [61], as well as the leaf veins being closer to this surface [62]. Therefore, shifting the distribution of leaf stomata to the abaxial surface may be a water conservation strategy.

The lower *f_gc_* minimizes the available stomatal area for transpirational water loss and *f_gc_* correlates positively with the leaf photosynthetic rate [63] and conductance [17]. The need for lower *f_gc_* is less urgent if water-stressed leaves are simply much smaller and thus conserve water by minimizing the plant’s transpirational surface. The negative correlation between the SD and SS is well-established within and across many species [19,64,65]. This tradeoff would not result in *f_gc_* plasticity, as we saw in water-stressed soybean leaves. However, the smaller stomata may be physiologically advantageous to water-deficit stressed plants, as smaller stomata can open and close more quickly [66,67,68,69] and can open at lower turgor pressure relative to larger stomata [70]. This means that plants in dry environments can quickly open their smaller stomata for photosynthesis after rainfall and rapidly re-close them as available water is depleted [71]. This results in a physiological benefit to leaves with smaller stomata even if the *f_gc_* remains consistent in water-stressed leaves. Similarly, a higher SD may not negatively affect the water status of stressed plants, as the fine control of the moment-to-moment size of the stomatal pore leads to no significant correlation between the SD and stomatal conductance [64,72]. In water-stressed soybean, the decrease in cell expansion ultimately led to a high SD/low SS stomatal phenotype, which has been associated with water-deficit stress [73] or more xeric environments [74], possibly because this phenotype is more sensitive to closure during drought conditions [75].

The vein density increase was common to both the maize genotype B73 × Mo17 and the soybean genotype IA3023. This response has not yet been characterized in water-stressed maize plants but has been reported in wheat [76]. In rice, another grass-type crop, the VD did not change in response to osmotic stress, but rice plants with a higher VD had a higher leaf photosynthesis and stomatal conductance rate [77]. In eudicots, such as cotton [78] and one soybean genotype investigated by de Sousa [79], the VD also increased in response to water deficit. Veins supply water to leaf tissues, hence the coordination of vein and SD is thought to balance the water supply via veins and loss via stomata [80]. The increased VD preserved the ratio of veins to stomata in the water-stressed maize and soybean and, thus, the supply and loss of water were balanced even when the SD increased, as in severely-stressed plants. This possibly ensured that drought-stressed tissues were sufficiently supplied with water. In plants where the leaf epidermis can evaporate more water than can be supplied by leaf venation, the stomata cannot remain open [81], which in our water-stressed maize and soybean plants would have prevented any growth and possibly led to plant death.

### 4.3. SPCH Expression Is Reduced in Response to Water Deficit in Maize and Soybean

We quantified the expression of *SPCH* homologues in the emerging leaf tissue of maize and soybean as a positive regulator of the entry division of protodermal cells into the stomatal development pathway [25,26]. Although maize and soybean are monocot and eudicot species, respectively, many stomatal development genes and proteins, including *SPCH*, are functionally conserved between these two plant types [82]. Therefore, we tested the hypothesis that the plasticity in stomatal development may be a result of a reduction in *SPCH* expression in both water-stressed maize and soybean plants. In Arabidopsis, the SPCH protein activity is reduced following drought stress [21]. Under water deficit conditions, *YDA* expression is upregulated [27] as its upstream repressor *AN3* is downregulated [83]. As YDA is a repressor of *SPCH*, *SPCH* expression is thus downregulated in water-stressed leaves [27,28]. The inhibition of SPCH activity or gene expression would therefore provide a mechanism to inhibit the differentiation of protodermal cells into stomata under water-deficit conditions. However, Jia et al. [84] observed the upregulation of *SPCH* gene expression in water-stressed plants. Therefore, the specific role of SPCH in response to water deficit has not yet been clearly established [31]. Moreover, other than in Arabidopsis [21], *SPCH* expression has not been quantified in conjunction with an examination of stomatal anatomy to substantiate its role in stomatal development plasticity in response to water deficit.

The SPCH family of transcription factors has been identified in maize and soybean [39], and we characterized expression of two of the three orthologs of *ZmSPCH* and three of the four orthologs of *GmSPCH*. All *SPCH* orthologs are significantly conserved in their functional domains (Supplementary Figure S12). Water deficit reduced the expression of only *ZmSPCH2* in maize and *GmSPCH3* in soybean (Figure 6). The *ZmSPCH2* expression was reduced in the 50% and 30% MWC treatments, and *GmSPCH3* expression reduced in the 30% MWC treatments, which matched the observed reduction in SI in both species. The different *SPCH* homologs may have evolved different promoter sequences, resulting in only certain gene copies being sensitive to, and thus downregulated by, osmotic stress. This has been observed in maize; *ZmSPCH1* expression was not responsive to mild drought in any region of developing maize leaves, whereas *ZmSPCH2* was downregulated in the drought-stressed regions of cell division and transition to cell elongation [37]. Xiang et al. [85] also found that the *ZmSPCH1* expression was not responsive to drought, although the *ZmSPCH2* expression was not quantified. In soybean, only *GmSPCH1* and *GmSPCH3* were still downregulated after 5 h of leaf dehydration, whereas the expression of other *SPCH* orthologs recovered by this time point [24]. *GmSPCH* expression may also be differentially regulated in different tissues and genotypes. *GmSPCH3* expression increased in the shoot apical meristem of water-stressed Harosoy and L62-364 soybean [86].

Different *SPCH* homologs may have neo-functionalized, as the bHLH transcription factor family to which this gene belongs has considerable functional diversity. In maize, neofunctionalization has been shown for the related *MUTE* transcription factor; despite the existence of at least two *MUTE* homologs in maize, the insertional mutation of just one (GRMZM2G417164) was sufficient to produce leaves without normal stomata [87]. *ZmSPCH1* has not yet been functionally characterized beyond its sequence-based annotation, so as yet there is no direct evidence that it promotes stomatal development. Similarly, the functional importance of the individual *SPCH* homologs in soybean leaves is presently unclear. A number of upstream regulators of the basal stomatal development pathway are likely also involved in the observed plasticity response to water stress [31,88]. *GTL1* has been implicated as a water-deficit responsive stomatal development regulator in *Arabidopsis* [22], and several other stomatal development transcription factors are responsive to the stress hormone ABA [89].

Our genetic and anatomical data suggest that the reduced stomatal development in response to water-deficit stress may be dependent on the reduced expression of *SPCH* in maize and soybean leaves, but only in response to severe water stress. In maize, the reduction in *ZmSPCH2* expression occurred in the 50% and 30% MWC grown leaves, whereas in soybean, *GmSPCH3* expression was reduced only in response to the 30% MWC treatment. As a result, *SPCH* may facilitate reduced differentiation of leaf stomatal development, resulting in reduced SI in maize and soybean. The *SPCH* downregulation, and the SI and *f_gc_* reduction did not occur in mild water deficits, consistent with other results from *Arabidopsis thaliana* [22], wheat [76], tomato [90], and soybean [79,91]. In these studies, the *f_gc_* or SI plasticity occurred in water-deficit treatments in which the MWC decreased by 70% [22], or when the leaf water potential decrease exceeded 0.5 MPa [76,91] relative to the control plants. Therefore, stomatal anatomical plasticity in plants may occur primarily or only as a response to severe water deficits.

## 5. Conclusions

**Figure 7:**
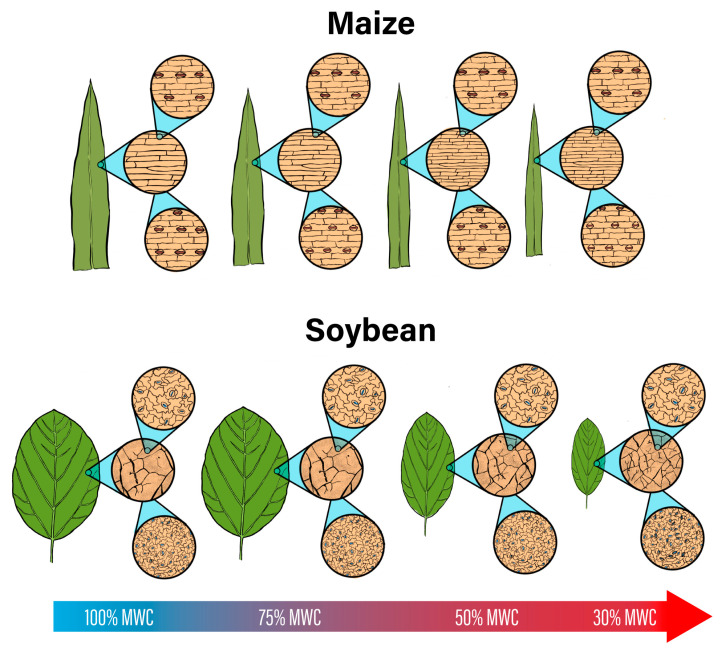
Contrasting leaf plasticity in maize and soybean leaves response to water-deficit stress, with the top and bottom row of images representing the adaxial and abaxial surfaces, respectively. Soybean leaves are greatly reduced in size due to water-deficit stress, leading to very small and dense epidermal cells. Relative to the degree of epidermal cell size reduction, SI reduction in soybean is not sufficient to minimize the stomatal population. Thus, while stomata are smaller, increased SD results in unchanged *f_gc_*. In maize, leaf and pavement cell size reduction occurs to a lesser degree, so the reduction in SI is sufficient to prevent a large increase in SD. Thus, smaller maize stomata result in lower *f_gc_* under water-deficit stress. Vein density is increased in both species.

In this study, we investigated the epidermal cell expansion, differentiation, and distribution responses to water deficit in maize and soybean. We observed epidermal changes in the water-stressed maize and soybean arising from cell differentiation and expansion processes, providing a mechanism to explain why the *f_gc_* reduction can occur in severely water-stressed leaves of some species. We show that the *f_gc_* is strongly and consistently decreased in response to the low MWC in maize but not soybean leaves. This was because, in water-stressed soybean leaves, large increases in the SD compensated for the reduction in the SS. Water stressed leaves of both species exhibited stomatal development plasticity, but only under severe water-deficit treatments, suggesting the plasticity response occurs only in extremely dry situations. This gives a model of distinct anatomical responses to water deficit in these genotypes (Figure 7). In both species, the SI reduction was attributable in part to the lower expression of certain *SPCH* orthologues. Our study demonstrates that the downregulation of *SPCH* expression may facilitate reduced stomatal development. However, the importance of the *f_gc_* plasticity to the water deficit response may differ between these maize and soybean genotypes. We observed several responses in soybean IA3023 that would have facilitated drought tolerance, such as thicker leaves, OA, and a small-but-dense stomatal phenotype, all of which may be more important environmental responses than stomatal plasticity.

Other genotypes of maize and soybean, possibly with different epidermal anatomy and/or water-stress sensitivity, may exhibit different anatomical responses to water deficit. Additionally, the physiological advantage of reduced or conserved *f_gc_* should be characterized in maize and soybean. Lastly, future studies should also investigate the activity of other stomatal development genes in inhibiting the stomatal development of water-stressed leaves [92,93,94,95,96,97].

## Data Availability

All data generated or analyzed in this study are presented in the article or its Supplementary Information.

## Supplementary Materials

The following supporting information can be downloaded at: https://www.mdpi.com/article/10.3390/life13020290/s1, Table S1: Results and diagnostics of the one-way ANOVA used in analyses of maize and soybean traits in the first experiment. Table S2: Results of the mixed linear model of maize and soybean traits in the second experiment. Table S3: Accession numbers and qPCR primer sequences of genes investigated in the current study. Table S4: Correlation results of leaf against water relations traits.; Figure S1: Response ratios for maize stomatal size, density, area fraction, index, abaxial-to-adaxial ratio, and leaf area in published literature. Figure S2: Response ratios for soybean stomatal size, density, area fraction, index, abaxial-to-adaxial ratio, and leaf area in published literature. Figure S3: Environmental conditions during the two experiments. Figure S4: Representative micrographs of maize and leaf epidermis in both experiments. Figure S5: Segmentation of maize leaves to quantify *SPCH* expression in response to water stress. Figure S6: Media water content in the first and second experiments. Figure S7: Average pre-watering media water content in the first and second experiments. Figure S8: Pavement cell size and abaxial-to-adaxial stomatal ratio in maize leaves. Figure S9: Pavement cell size and abaxial-to-adaxial stomatal ratio in soybean leave. Figure S10: Ratio of stomatal to vein density in maize and soybean leaves. Figure S11: Relationships between stomatal density and size. Figure S12: Amino acid sequence conservation between *SPCH* orthologs in *Arabidopsis thaliana*, *Zea mays*, and *Glycine max*.
