# Supplementary material for: Different Leaf Anatomical Responses to Water Deficit in Maize and Soybean
Source: Life (Basel). 2023 Jan 20;13(2):290. doi: 10.3390/life13020290 (PMC9966819; doi:10.3390/life13020290)
Supplement: Supplementary file 1 [file life-13-00290-s001.zip › life-2127577-supplementary.pdf]

Supplementary Material

Supplementary Table S1 Results and diagnostics of the one-way ANOVA used in analysis of maize and soybean epidermal and plant traits in the first experiment.

				One-way ANOVA assumption tests		One-way ANOVA results	
Trait	Leaf surface	Species	Leaf	Shapiro-Wilk test of normality	Bartlett's constant variances test	*F*	*P*
Stomatal density	Adaxial	Maize	7	0.490	0.755	0.591	0.630
		Soybean	4	0.544	0.222	0.325	0.807
			5	0.920	0.155	2.14	0.136
	Abaxial	Maize	7	0.408	0.580	3.77	0.032
		Soybean	4	0.271	0.539	7.33	0.003
			5	0.998	0.359	16.0	<0.001
Stomatal size	Adaxial	Maize	7	0.904	0.827	15.4	<0.001
		Soybean	4	0.063	0.408	9.26	0.005
			5	0.002†	0.056	8.07	0.002
	Abaxial	Maize	7	0.102	0.249	19.8	<0.001
		Soybean	4	0.046†	0.331	15.5	<0.001
			5	0.979	0.540	43.3	<0.001
Stomatal area fraction	Adaxial	Maize	7	0.943	0.880	13.4	<0.001
		Soybean	4	0.205	0.162	1.64	0.221
			5	0.129	0.022‡	3.50	0.068
	Abaxial	Maize	7	0.574	0.669	9.59	<0.001
		Soybean	4	0.447	0.682	4.71	0.015
			5	0.697	0.688	3.83	0.030
Stomatal index	Adaxial	Maize	7	0.401	0.824	11.6	<0.001
		Soybean	4	0.533	0.017‡	36.7	<0.001
			5	1.000	0.306	5.51	0.009

	Abaxial	Maize	7	0.788	0.645	0.933	0.448
		Soybean	4	0.760	0.428	1.32	0.302
			5	0.572	0.785	1.65	0.217
Pavement cell density	Adaxial	Maize	7	0.371	0.874	3.72	0.033
		Soybean	4	0.332	0.698	6.53	0.004
			5	0.005 †	0.036 ‡	5.58	0.021
	Abaxial	Maize	7	0.679	0.473	5.49	0.009
		Soybean	4	0.105	0.184	8.85	0.001
			5	0.538	0.141	19.0	<0.001
Pavement cell size	Adaxial	Maize	7	0.383	0.766	3.36	0.045
		Soybean	4	0.992	0.558	5.12	0.011
			5	0.822	0.419	14.5	<0.001
	Abaxial	Maize	7	0.249	0.741	5.49	0.009
		Soybean	4	0.265	0.309	7.14	0.003
			5	0.632	0.435	13.8	<0.001
Abaxial: adaxial stomatal ratio		Maize	7	0.747	0.428	1.43	0.272
		Soybean	4	0.498	0.206	2.12	0.138
			5	0.764	0.015‡	1.26	0.322
Relative water content		Maize	7	0.010†	0.008 ‡	3.26	0.079
		Soybean	5	0.609	0.726	27.2	<0.001
Osmotic potential		Maize	7	0.446	0.039 ‡	6.77	0.054
		Soybean	4	0.091	0.894	1.11	0.375
Osmotic potential at full turgor		Maize	7	0.380	0.041 ‡	6.75	0.268
		Soybean	4/5	0.036 †	0.468	1.25	0.325

† indicates Shapiro-Wilk test *P*-values <0.05, and thus violations of the assumption of normality. In these instances, the data was Box-Cox transformed (λ value of 0) and subsequent values are from the transformed data. ‡ indicates Bartlett's test *P*-values <0.05, wherein variances are unequal within groups and Welch's one-way ANOVA tests and Games-Howell post-hoc tests are used.

Supplementary Table S2 Results of the mixed linear model used in analysis of maize and soybean epidermal and plant traits in the second experiment.

			Mixed linear model results		
Trait	Leaf surface	Species	Shapiro-Wilk test of normality	*F*	$P_{bonferroni}$
Stomatal density	Adaxial	Maize	0.107	1.84	0.176
		Soybean	0.011[†]	2.16	0.124
	Abaxial	Maize	0.239	5.19	0.010
		Soybean	0.956	26.1	<0.001
Stomatal size	Adaxial	Maize	0.312	19.7	<0.001
		Soybean	0.628	46.8	<0.001
	Abaxial	Maize	0.133	19.8	<0.001
		Soybean	0.259	55.9	<0.001
Stomatal pore index	Adaxial	Maize	0.863	8.02	<0.001
		Soybean	0.750	0.333	0.802
	Abaxial	Maize	0.673	5.68	0.007
		Soybean	0.547	5.600	0.006
Stomatal index	Adaxial	Maize	0.311	6.69	0.003
		Soybean	0.839	29.9	<0.001
	Abaxial	Maize	0.514	12.8	<0.001
		Soybean	0.293	8.91	<0.001
Pavement cell density	Adaxial	Maize	0.200	16.6	<0.001
		Soybean	0.001[†]	53.4	<0.001
	Abaxial	Maize	0.933	17.4	<0.001
		Soybean	0.001[†]	81.7	<0.001
Pavement cell size	Adaxial	Maize	0.005[†]	13.0	<0.001
		Soybean	0.093	61.6	<0.001
	Abaxial	Maize	0.423	13.8	<0.001

		Soybean	0.055	85.2	<0.001
Total leaf stomata	Adaxial	Maize	0.642	28.8	<0.001
		Soybean	0.493	42.4	<0.001
	Abaxial	Maize	0.992	18.8	<0.001
		Soybean	0.873	27.8	<0.001
Total leaf pavement cells	Adaxial	Maize	0.423	11.9	<0.002
		Soybean	0.846	28.5	<0.001
	Abaxial	Maize	0.986	16.8	<0.001
		Soybean	0.170	35.6	<0.001
Abaxial: adaxial stomatal ratio		Maize	0.373	4.90	0.012
		Soybean	0.582	11.8	<0.001
Leaf area		Maize	<0.001[†]	52.5	<0.001
		Soybean	0.349	87.5	<0.001
Vein density		Maize	0.764	30.71	<0.001
		Soybean	0.051	48.88	<0.001
Adaxial stomatal: vein density		Maize	0.841	2.294	0.113
		Soybean	0.077	3.405	0.037
Abaxial stomatal: vein density		Maize	0.723	5.594	0.007
		Soybean	0.284	0.155	0.925
Total stomatal: vein density		Maize	0.730	3.531	0.035
		Soybean	0.246	0.811	0.503
Specific leaf weight		Maize	0.626	3.03	0.057
		Soybean	0.917	24.8	<0.001
Leaf relative water content		Maize	0.419	392	<0.001
		Soybean	0.427	120	<0.001
Leaf osmotic potential		Maize	0.088	257	<0.001

	Soybean	0.523	114	<0.001
Leaf osmotic potential at full turgor	Maize	0.367	18.9	<0.001
	Soybean	0.883	69.9	<0.001

Mixed linear models used water-deficit treatments as fixed factors and blocks as random effects. *F*-scores and *P*-values for the overall model are shown. † indicate Shapiro-Wilk test *P*-values <0.05, and thus violations of the assumption of normality. In these instances, the data was Box-Cox transformed (λ value of 0) and the *F* and *P*-values are for the transformed data.

Supplementary Table S3 Accession numbers of genes investigated in the current study and their qRT-PCR primer sequences

Gene	Accession number	Primer sequence
ZmSPCH1	GRMZM2G045109	F CGTAGGGTTGTTGTTGGCTTA
		R GCTGCTGCTCTGCTTGTTACTA
ZmSPCH2	GRMZM2G085751	F AGCAATCATCACATGGCGGA
		R CAAGAGGTCGTCGGTATCGG
ZmTUB2	GRMZM2G334899	F CTACCTCACGGCATCTGCTATGT
		R GTCACACACACTCGACTTCACG
GmSPCH1	Glyma04g238400	F CTCCACACTCTCCACCACTTTT
		R CCAGCGAAGGGAAGTACCG
GmSPCH2	Glyma06g125500	F TCGCAGGTTTCACGGAGAAG
		R GAGGAGGGAAATCGGTGGTG
GmSPCH3	Glyma13g08740	F GTGCTGAAGAACTCGCTCAA
	now Glyma13g040100	R AAACAACACACTAAAATGGTCTGAT
GmACT11	Glyma02g10170	F ATCTTGACTGAGCGTGGTTATTCC
		R GCTGGTCCTGGCTGTCTCC

All primers are described in the 5’ to 3’ direction

Supplementary Table S4 Correlation results of leaf against water relations traits

Trait		Experiment	Species	Correlation results	
				Pearson's *r*	*P*
Adaxial SD	RWC	1	Maize	0.300	0.259
			Soybean	-0.550	0.012
		2	Maize	-0.305	0.115
			Soybean	-0.558	<0.001
	Ψ_{100}	1	Maize	0.070	0.797
			Soybean	-0.187	0.429
		2	Maize	-0.119	0.548
			Soybean	-0.260	0.151
Abaxial SD	RWC	1	Maize	-0.236	0.379
			Soybean	-0.869	<0.001
		2	Maize	-0.468	0.012
			Soybean	-0.879	<0.001
	Ψ_{100}	1	Maize	-0.195	0.469
			Soybean	0.159	0.504
		2	Maize	0.273	0.160
			Soybean	-0.551	0.001
Adaxial SS	RWC	1	Maize	0.462	0.071
			Soybean	0.584	0.007
		2	Maize	0.780	<0.001
			Soybean	0.645	<0.001
	Ψ_{100}	1	Maize	0.022	0.935
			Soybean	-0.486	0.030
		2	Maize	0.131	0.506
			Soybean	0.450	0.011

Abaxial SS	RWC	1	Maize	0.563	0.023
			Soybean	0.811	<0.001
		2	Maize	0.713	<0.001
			Soybean	0.749	<0.001
	Ψ_{100}	1	Maize	0.007	0.980
			Soybean	-0.317	0.174
		2	Maize	0.225	0.249
			Soybean	0.551	0.002
Adaxial f_{gc}	RWC	1	Maize	0.556	0.025
			Soybean	0.052	0.828
		2	Maize	0.646	<0.001
			Soybean	0.024	0.900
	Ψ_{100}	1	Maize	0.087	0.749
			Soybean	-0.496	0.026
		2	Maize	0.087	0.659
			Soybean	0.099	0.598
Abaxial f_{gc}	RWC	1	Maize	0.538	0.032
			Soybean	0.275	0.240
		2	Maize	0.442	0.019
			Soybean	-0.461	0.010
	Ψ_{100}	1	Maize	-0.169	0.530
			Soybean	-0.306	0.190
		2	Maize	0.365	0.056
			Soybean	-0.270	0.149
Adaxial SI	RWC	1	Maize	0.484	0.030
			Soybean	0.613	0.004
		2	Maize	0.608	<0.001
			Soybean	0.819	<0.001

	Ψ_{100}	1	Maize	0.310	0.184
			Soybean	-0.208	0.379
		2	Maize	-0.043	0.830
			Soybean	0.758	<0.001
Abaxial SI	RWC	1	Maize	0.159	0.556
			Soybean	0.173	0.466
		2	Maize	0.714	<0.001
			Soybean	0.664	<0.001
	Ψ_{100}	1	Maize	-0.286	0.283
			Soybean	0.068	0.777
		2	Maize	0.019	0.926
			Soybean	0.585	<0.001
Adaxial PS	RWC	1	Maize	0.154	0.568
			Soybean	0.870	<0.001
		2	Maize	0.716	<0.001
			Soybean	0.922	<0.001
	Ψ_{100}	1	Maize	0.175	0.518
			Soybean	-0.015	0.951
		2	Maize	0.031	0.874
			Soybean	0.742	<0.001
Abaxial PS	RWC	1	Maize	0.253	0.344
			Soybean	0.791	<0.001
		2	Maize	0.757	<0.001
			Soybean	0.902	<0.001
	Ψ_{100}	1	Maize	0.126	0.642
			Soybean	-0.073	0.759
		2	Maize	-0.204	0.298
			Soybean	0.686	<0.001

Total adaxial cells	RWC	1	Maize	0.517	0.040
			Soybean	0.674	0.001
		2	Maize	0.665	<0.001
			Soybean	0.777	<0.001
	Ψ_{100}	1	Maize	0.209	0.437
			Soybean	-0.230	0.330
		2	Maize	-0.191	0.331
			Soybean	0.668	<0.001
Total abaxial cells	RWC	1	Maize	0.501	0.048
			Soybean	0.694	<0.001
		2	Maize	0.698	<0.001
			Soybean	0.836	<0.001
	Ψ_{100}	1	Maize	0.261	0.328
			Soybean	-0.198	0.402
		2	Maize	-0.024	0.903
			Soybean	0.739	<0.001
Stomatal ratio	RWC	1	Maize	-0.219	0.354
			Soybean	-0.279	0.234
		2	Maize	-0.245	0.209
			Soybean	-0.647	<0.001
	Ψ_{100}	1	Maize	-0.174	0.462
			Soybean	0.333	0.151
		2	Maize	0.349	0.068
			Soybean	-0.530	0.002
LA	RWC	1	Maize	0.556	0.025
			Soybean	0.806	<0.001
		2	Maize	0.839	<0.001
			Soybean	0.918	<0.001

	Ψ_{100}	1	Maize	0.301	0.258
			Soybean	-0.216	0.361
		2	Maize	-0.085	0.666
			Soybean	0.781	<0.001
SLW	RWC	1	Maize	-0.087	0.748
			Soybean	0.264	0.261
		2	Maize	0.284	0.143
			Soybean	-0.536	0.002
	Ψ_{100}	1	Maize	0.130	0.632
			Soybean	0.018	0.940
		2	Maize	0.029	0.883
			Soybean	-0.847	<0.001
VD	RWC	2	Maize	-0.683	<0.001
			Soybean	-0.865	<0.001
	Ψ_{100}	2	Maize	-0.171	0.386
			Soybean	-0.652	<0.001
AdSD:VD	RWC	2	Maize	0.309	0.110
			Soybean	0.492	0.005
	Ψ_{100}	2	Maize	0.051	0.795
			Soybean	0.354	0.051
AbSD:VD	RWC	2	Maize	0.031	0.876
			Soybean	0.149	0.433
	Ψ_{100}	2	Maize	0.528	0.004
			Soybean	0.054	0.777
TotalSD:VD	RWC	2	Maize	0.195	0.320
			Soybean	0.326	0.079
	Ψ_{100}	2	Maize	0.415	0.028
			Soybean	0.190	0.314

RWC	Ψ_{100}	1	Maize	-0.101	0.711
			Soybean	-0.196	0.409
		2	Maize	-0.137	0.487
			Soybean	0.720	<0.001

RWC- leaf relative water content, Ψ_{100} – leaf osmotic potential at full turgor

Supplementary Figures

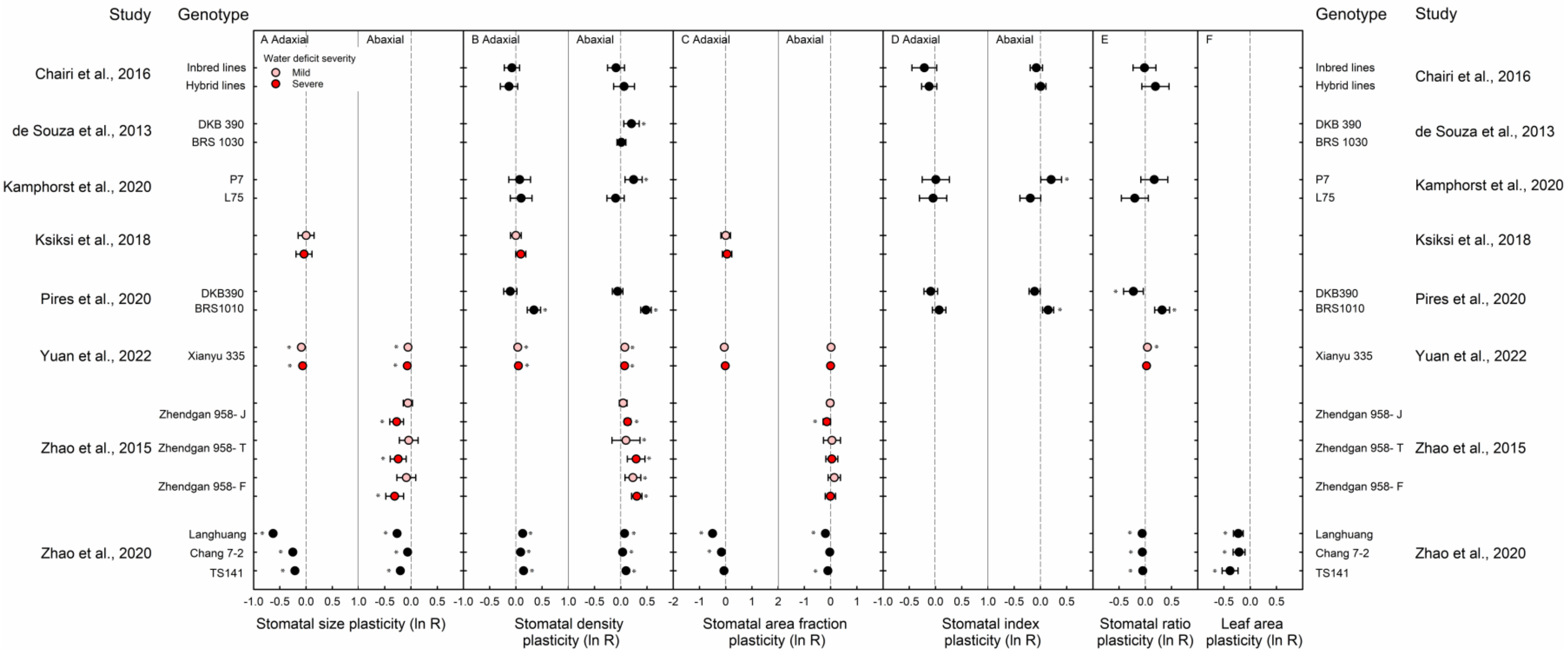

Supplementary Figure S1. Compilation of response ratios (ln R) for stomatal size (A), density (B), area fraction (C), index (D), abaxial: adaxial stomatal ratio (E), and leaf area (F) in published literature investigating epidermal plasticity in water-stressed maize leaves. Data are shown as individual comparisons against well-watered controls, and error bars represent 95% confidence intervals. If a particular genotype was subjected to several levels of water-deficit treatment, data points were colored pink for milder stresses and red for more severe treatments. Dashed lines represent a ln R value of 0; if error bars do not cross this line, the plasticity value is significant. Statistical significance is also indicated by *. Data are shown for studies with valid experimental methodology such that leaves sampled were known to have developed under water-deficit conditions.

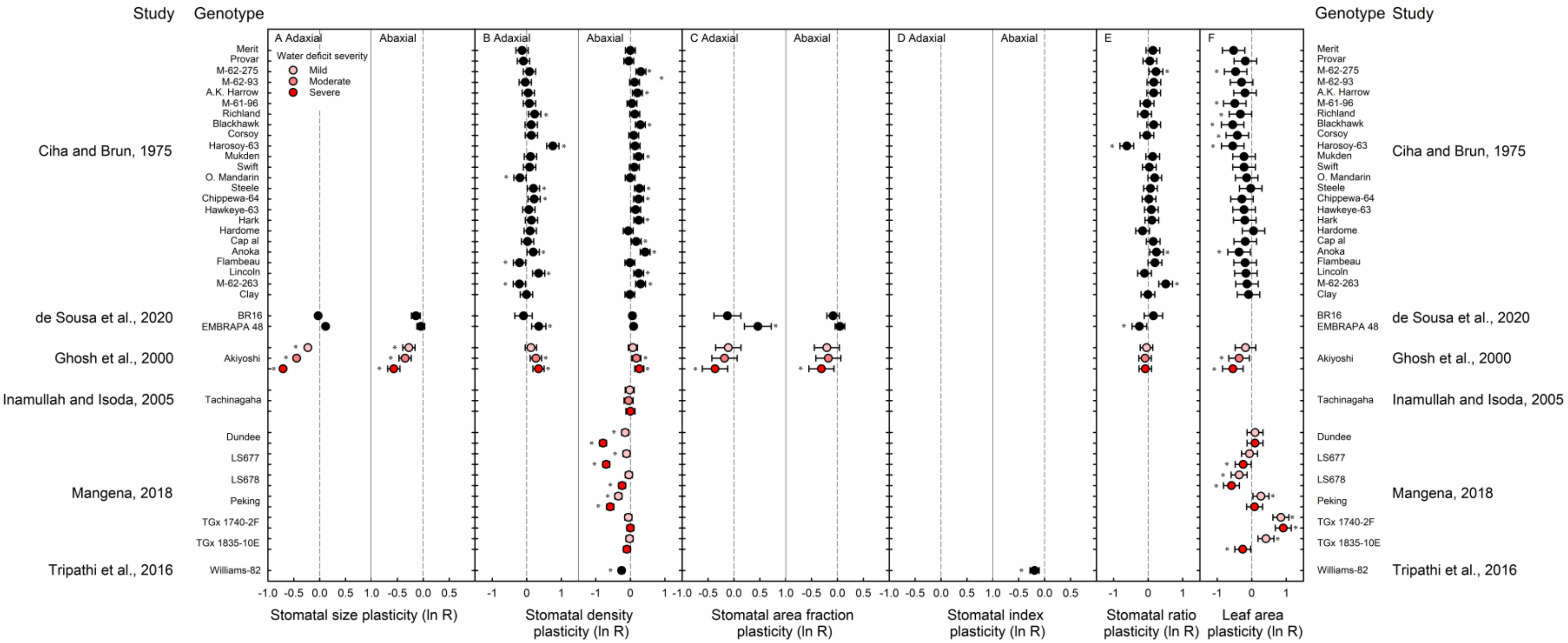

Supplementary Figure S2. Compilation of response ratios (ln R) for stomatal size (A), density (B), area fraction (C), index (D), abaxial: adaxial stomatal ratio (E), and leaf area (F) in published literature investigating epidermal plasticity in water-stressed soybean leaves. Data are shown as individual comparisons against well-watered controls, and error bars represent 95% confidence intervals. If a particular genotype was subjected to several levels of water-deficit treatment, data points were colored light pink for mild stress, pink for moderate stress, and red for the most severe treatments. Dashed lines represent a ln R value of 0; if error bars do not cross this line, the plasticity value is significant. Statistical significance is also indicated by *. Data are shown for studies with valid experimental methodology such that leaves sampled were known to have developed under water-deficit conditions.

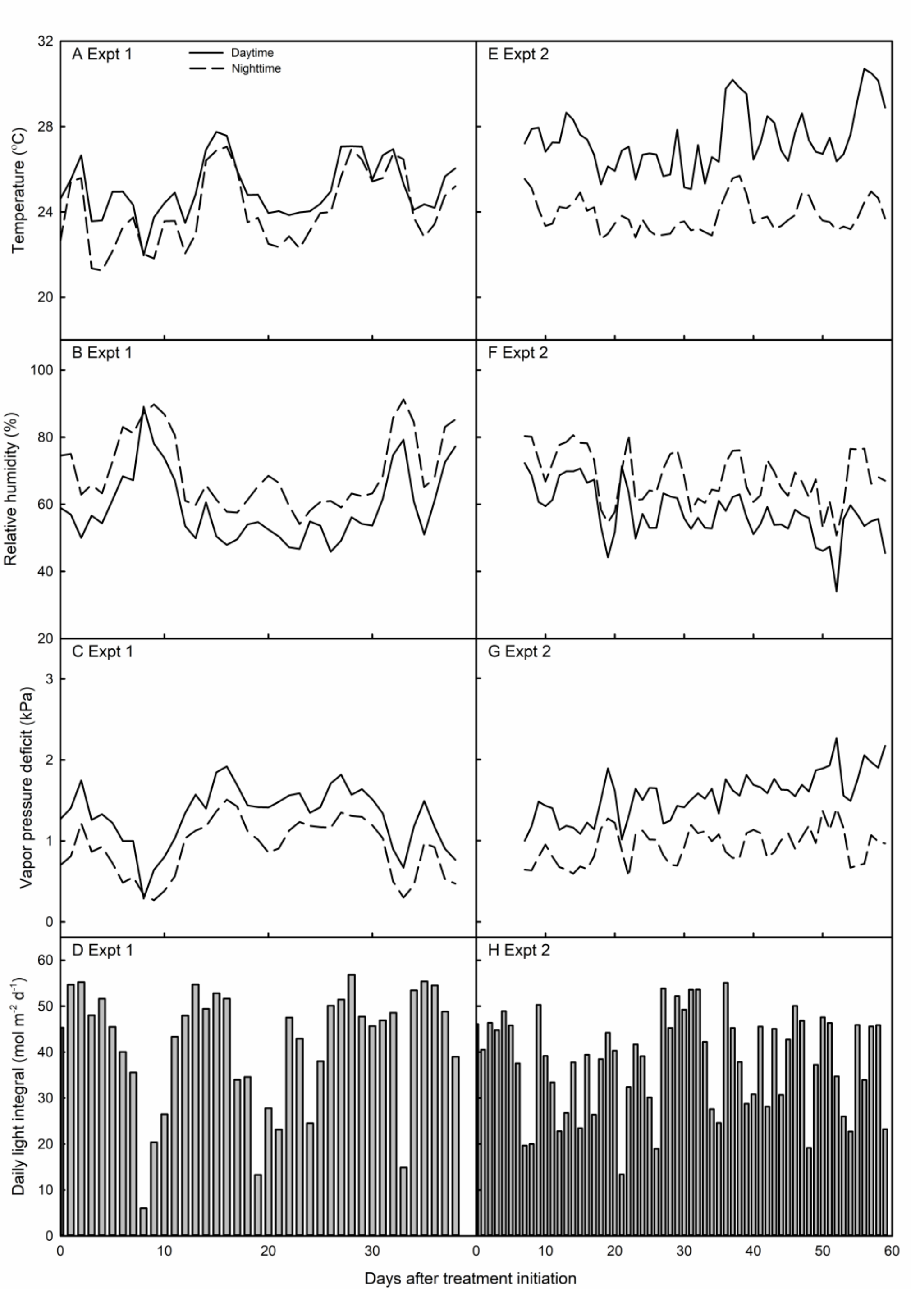

Supplementary Figure S3. Environmental conditions during the first (A–D) and second water deficit experiments (E–H). Data shown are average daytime and nighttime temperature (A, E), relative humidity (B, F), and vapor pressure deficit (C, G). The daily light integral for each experimental day is also shown (D, H), including supplemental lighting delivered inside the greenhouse to maintain at least a 12-hour photoperiod.

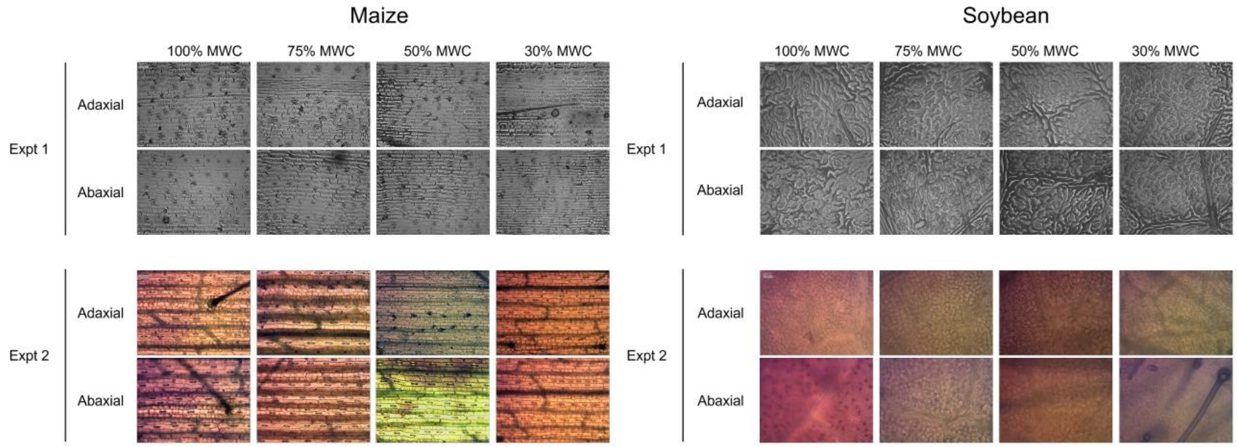

Supplementary Figure S4. Representative epidermal micrographs of maize and soybean leaves from the first and second experiments. Impressions were made with cyanoacrylate droplets pressed onto a glass slide in the first experiment, whereas in the second experiment, sections of tissue were cleared and stained for imaging. For further details, refer to the Materials and Methods, 2.4- Anatomical traits. Where parts of an image are out of focus, a second image from the same plane of view was taken with adjusted focus to enable accurate cell counts despite the heterogeneity in the leaf surface.

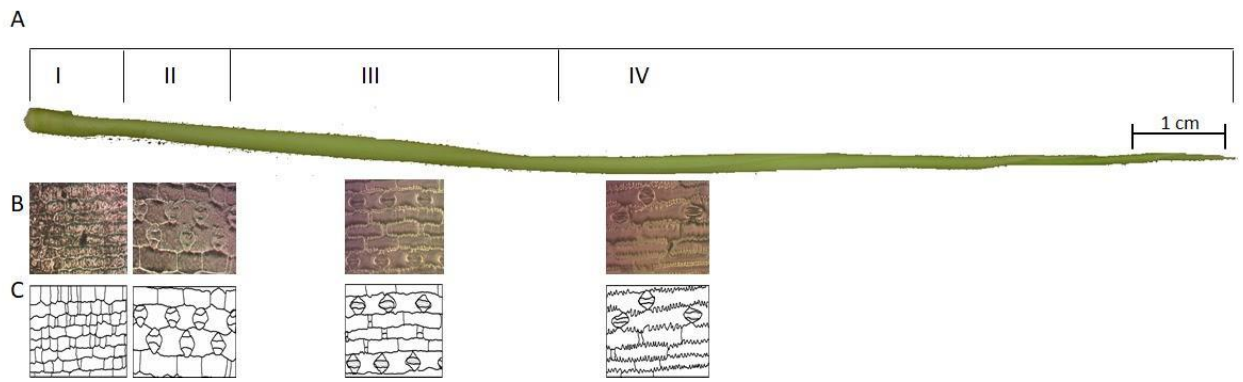

Supplementary Figure S5. Segmentation of maize leaves used to quantify *SPCH* expression in response to water stress. Maize leaf 8 was removed from within the leaf sheath and divided into four segments (A), which showed epidermal cells in different stages of cell differentiation (B, C). Segment I, 2 from 0 to 1 cm above the leaf base, consisted of undifferentiated protodermal cells. Segment II, from 1–2 cm above the leaf base, and segment III, from 2–6 cm above the leaf base, consisted of partially differentiated stomatal complexes and pavement cells. Segment IV, from 6 cm to the remaining length of the leaf, consisted of mature epidermal cells with established identity. Segments I and IV were used to quantify *ZmSPCH* expression.

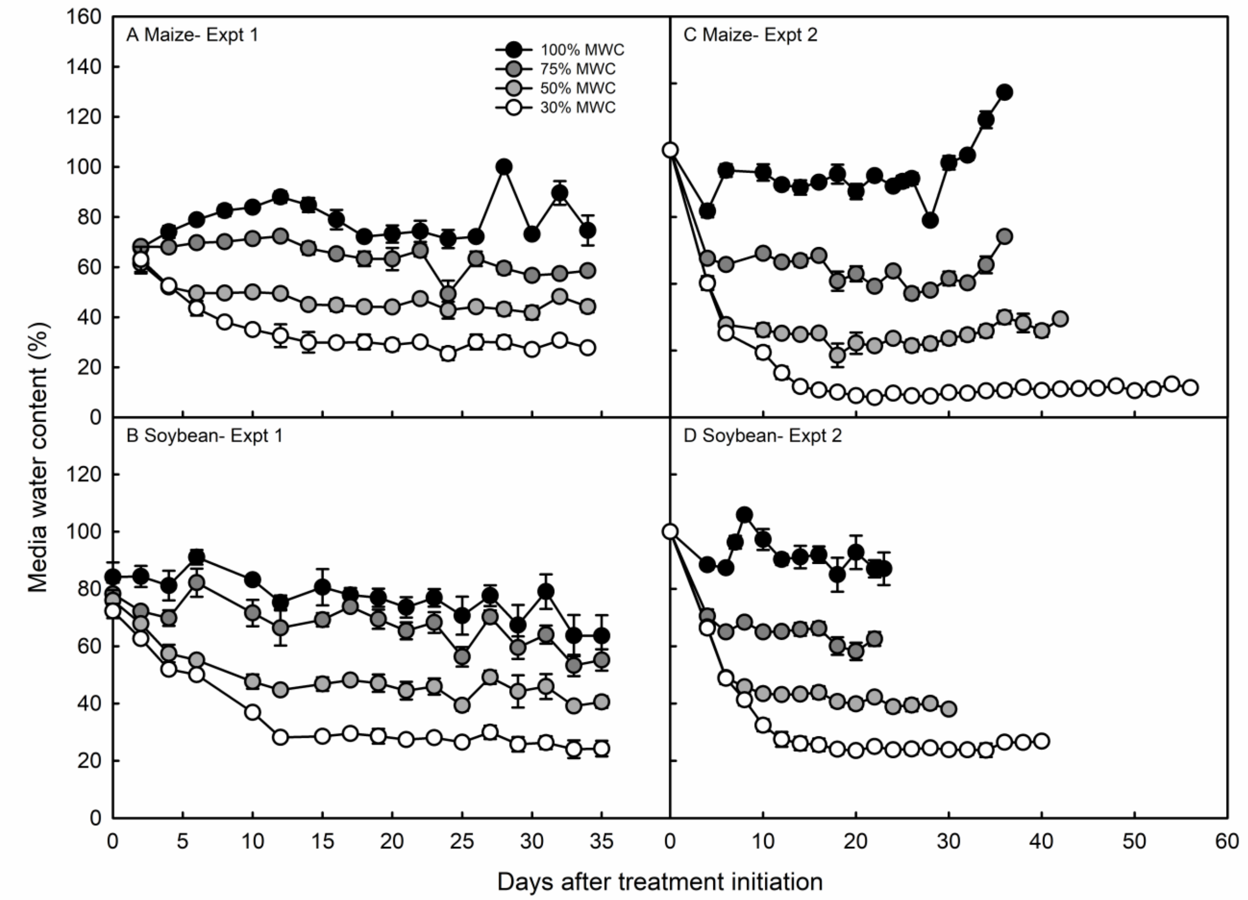

Supplementary Figure S6. Media water content (MWC) in the first (A, B) and second experiments (C, D) for maize and soybean plants subjected to different water deficit treatments. At each time point, MWC was averaged between the values before and after irrigation. Data are presented as means ± standard error for 5 soybean and 4 maize plants per species per treatment in the first experiment, and 8 plants per species per treatment in the second experiment.

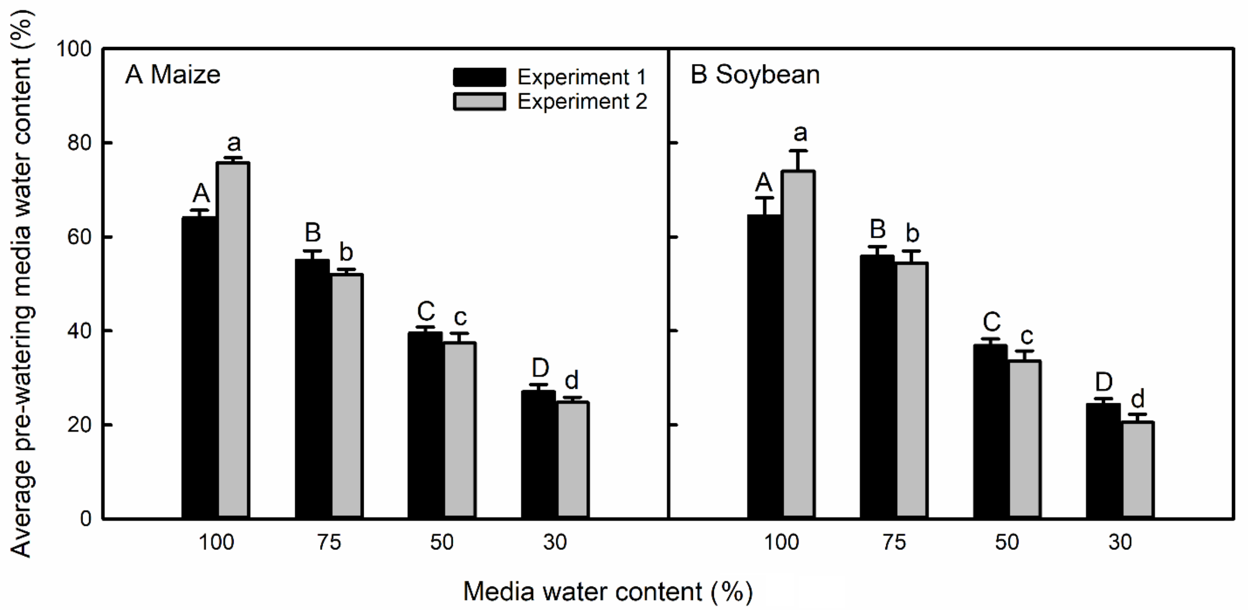

Supplementary Figure S7. Comparison of the average pre-watering media water content (MWC) in experiments 1 and 2 for maize (A) and soybean (B) plants. Data are shown as means ± standard errors for n= 4 maize plants and 5 soybean plants in the first experiment and n= 8 in the second experiment. Within each experiment, treatment groups are compared using a one-way ANOVA (experiment 1), or a mixed linear model using water-deficit treatments as the fixed factor and blocks as the random effect (experiment 2). Different letters above the columns indicate statistically significant differences between treatments within experiment 1 (uppercase) and experiment 2 (lowercase).

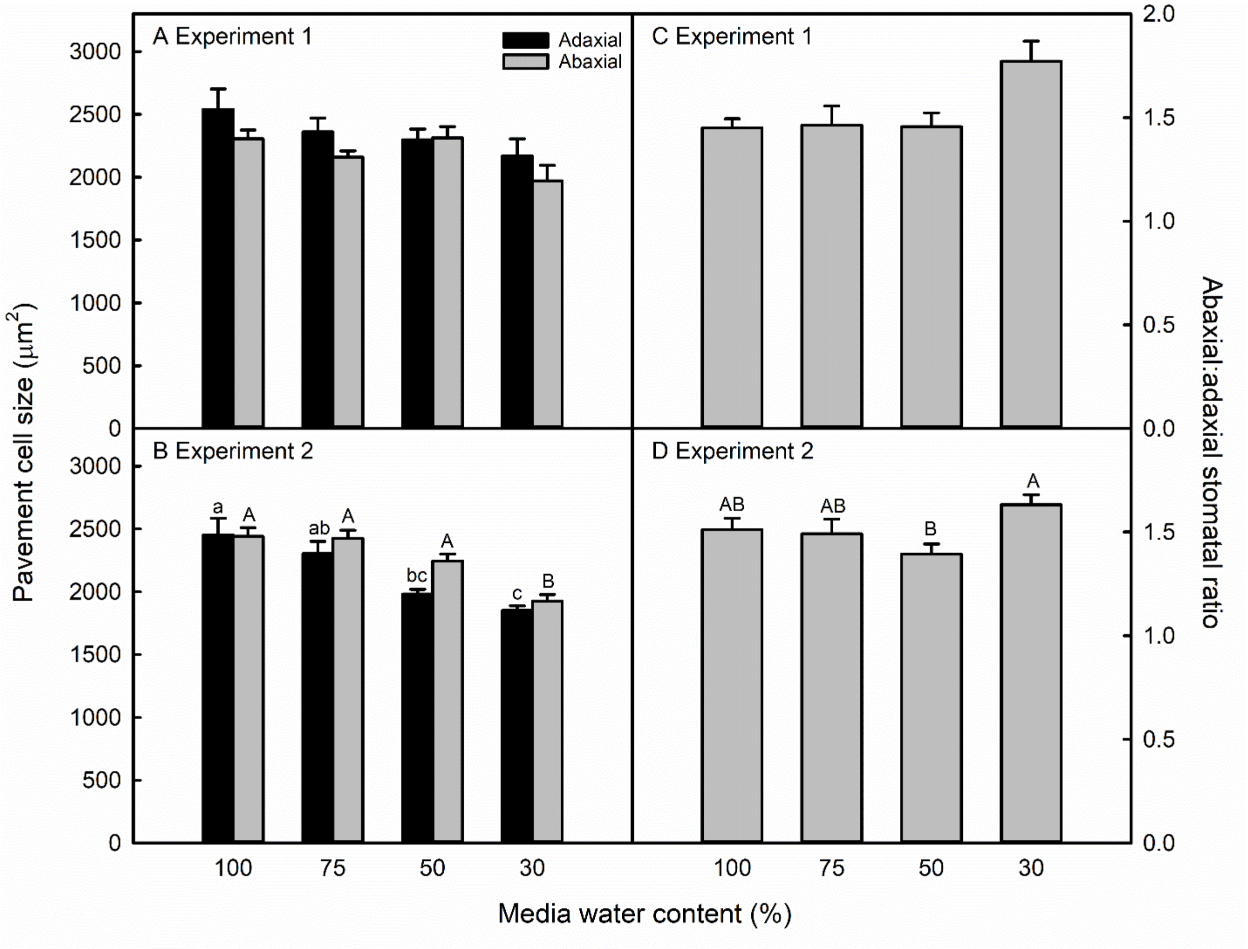

Supplementary Figure S8. Pavement cell size (A, B) and the abaxial-to-adaxial stomatal (C, D) ratio in maize leaves exposed to different media water content treatments in the first (A, C) and second (B, D) experiments. Different letters above the columns indicate statistically significant differences between treatments within adaxial (lowercase) and abaxial (uppercase) leaf surfaces. In the first experiment, treatment groups were compared with a one-way ANOVA and post-hoc Tukey or Games-Howell test, depending on the equivalence of variance between treatment groups. In the second experiment, treatment groups were compared with a mixed linear model and post-hoc test with Bonferroni correction. Data are shown as means ± standard errors for $n = 4$ in the first experiment and $n = 8$ in the second experiment.

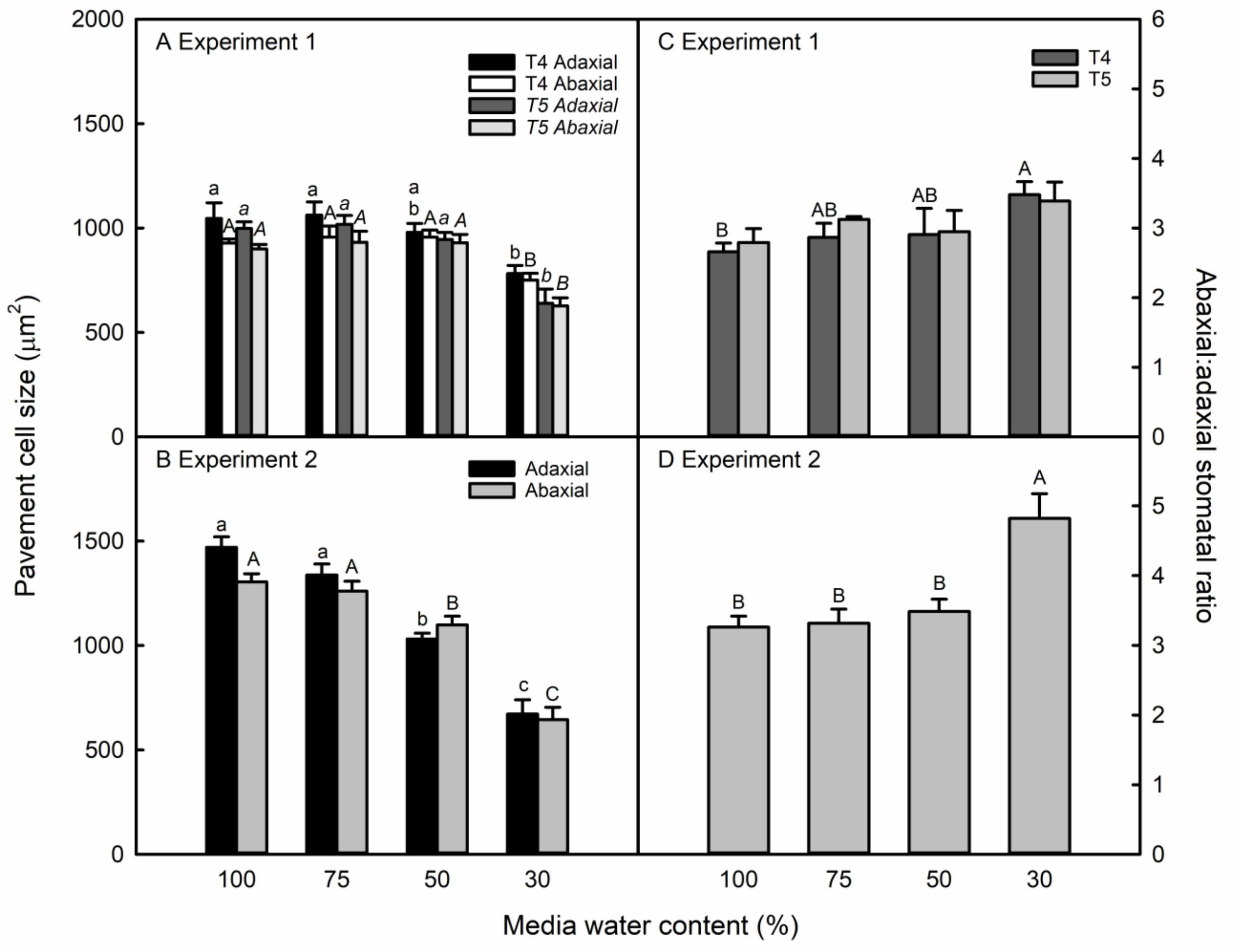

Supplementary Figure S9. Pavement cell size (A, B) and the abaxial-to-adaxial stomatal ratio (C, D) in soybean leaves exposed to different media water content treatments in the first (A, C) and second (B, D) experiments. Different letters above the columns indicate statistically significant differences between treatments within adaxial (lowercase) and abaxial (uppercase) leaf surfaces. In the first experiment, treatment groups were compared within the fourth (T4) or fifth trifoliates (T5) using a one-way ANOVA and post-hoc Tukey or Games-Howell test, depending on the equivalence of variance between treatment groups. In the second experiment, treatment groups were compared with a mixed linear model and post-hoc test with Bonferroni correction. Data are shown as means ± standard errors for *n*= 5 in the first experiment and *n*= 8 in the second experiment.

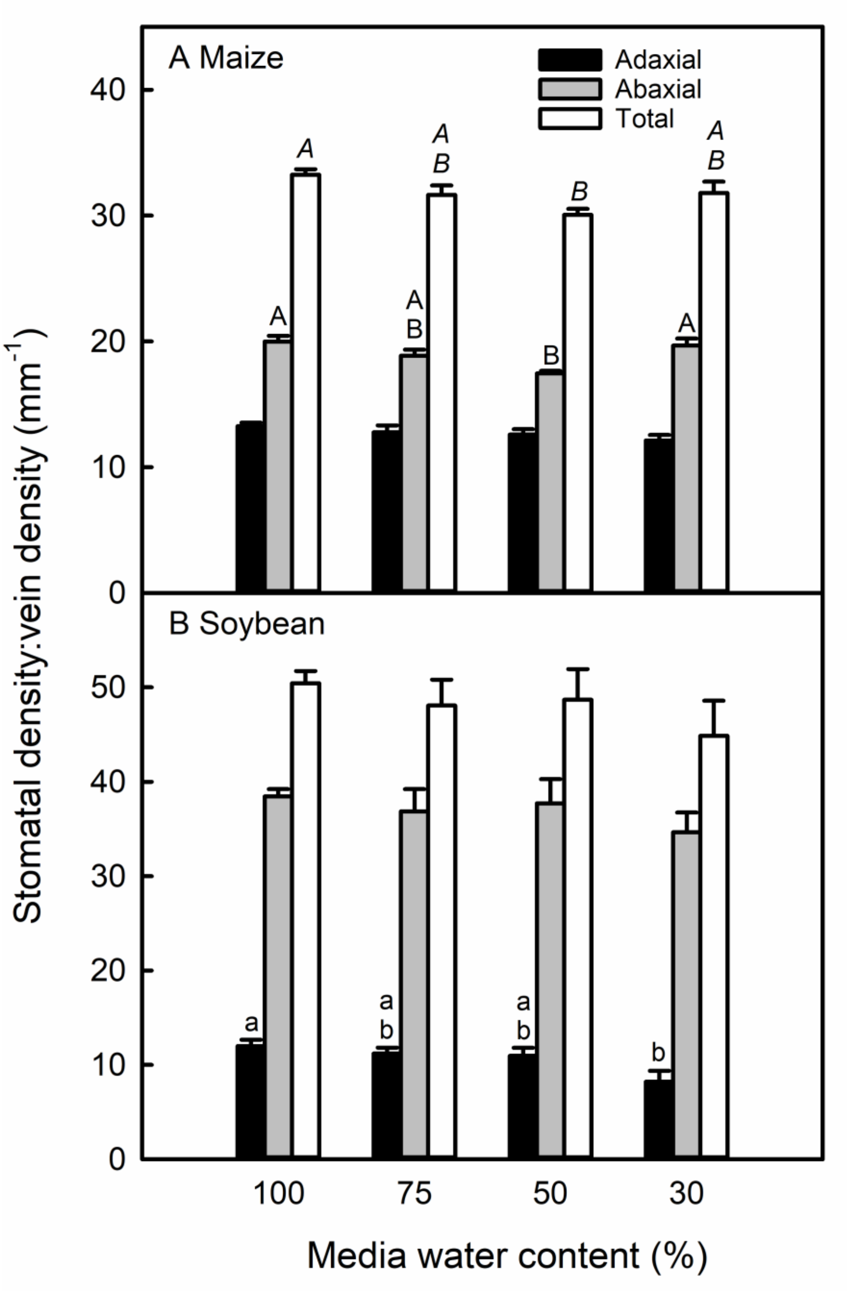

Supplementary Figure S10. The ratio of stomatal density to vein density ratio within and across leaf surfaces in maize (A) and soybean (B) exposed to different media water content treatments in the second experiment. Different letters above the columns indicate statistically significant differences between treatments within adaxial (lowercase), abaxial (uppercase), and across both (italicized) leaf surfaces. Treatment groups were compared with a mixed linear model and post-hoc test with Bonferroni correction. Data are shown as means ± standard errors for *n*= 8.

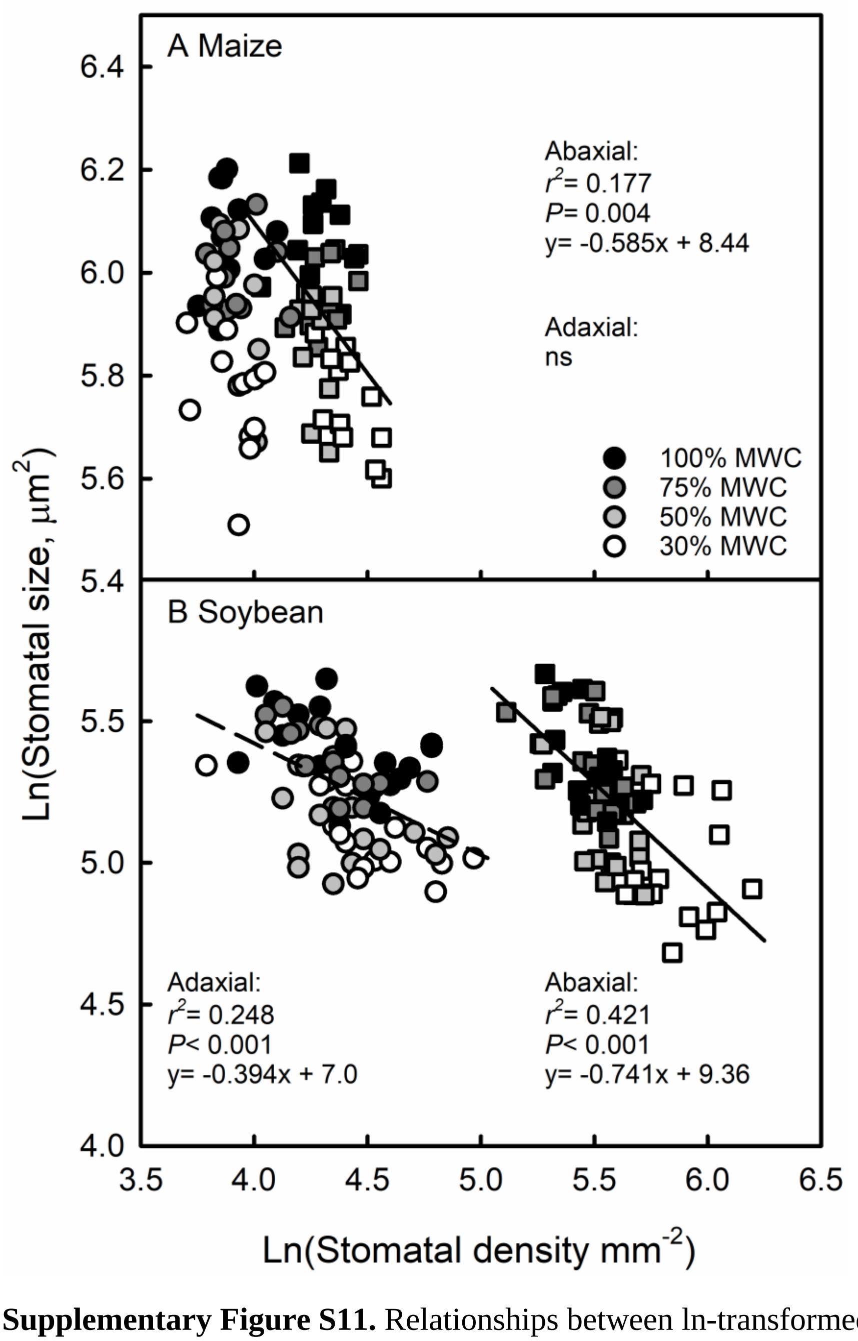

Supplementary Figure S11. Relationships between ln-transformed stomatal density and stomatal size in maize (A) and soybean (B) plants in both experiments. Data are shown for adaxial (circles and dashed lines) and abaxial (squares and solid lines) leaf surfaces. Regression lines are fitted through all data in each leaf surface, when the overall relationship was statistically significant (P< 0.05). r^2 values and regression equations for ln-transformed data are shown.

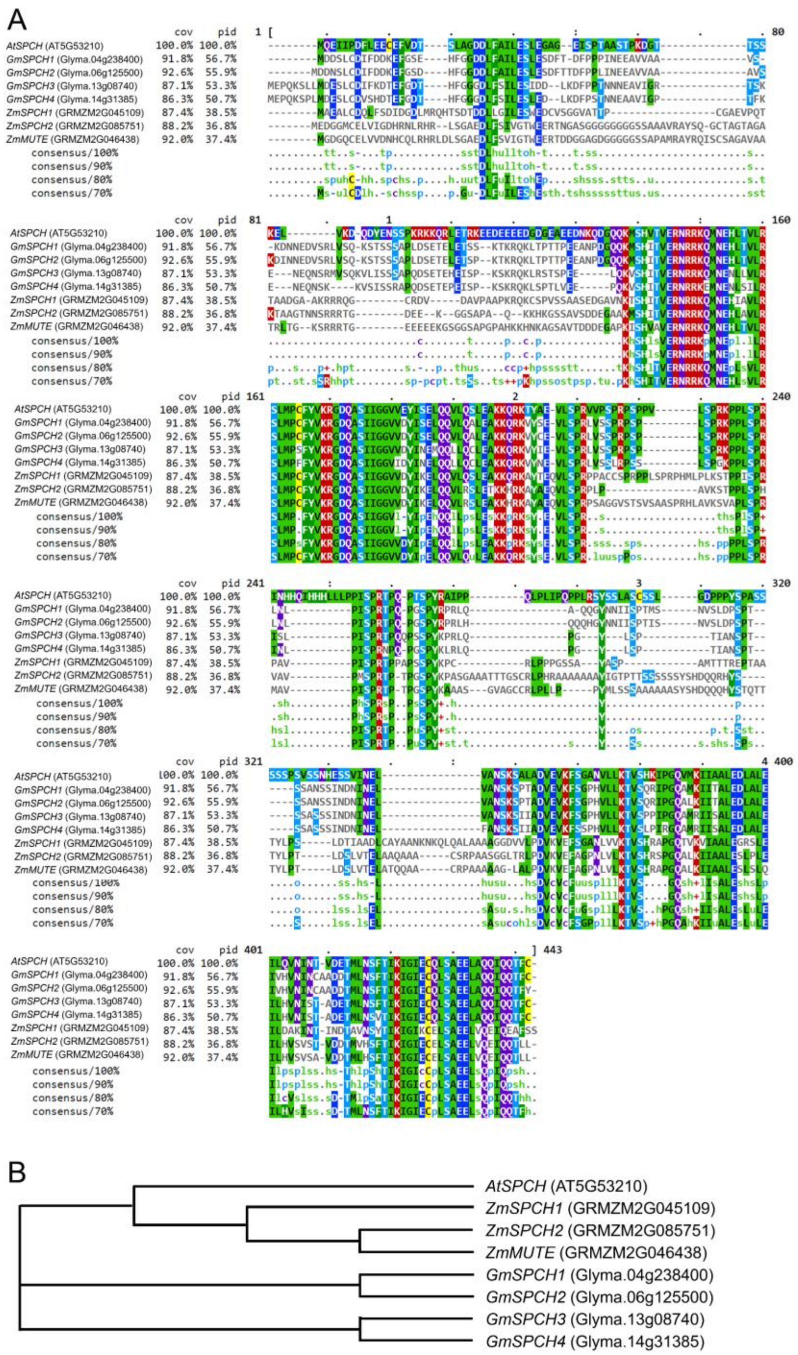

Supplementary Figure S12. Amino acid sequence conservation between orthologs of *SPCH* in *Arabidopsis thaliana*, *Zea mays*, and *Glycine max* (A). GRMZM2G046438 was included as a *SPCH* orthologue identified by (Ran et al., 2013) but is now classified as an orthologue of *MUTE*. Sequence alignment was performed in ClustalW, which also generated the phylogenetic relationships based on sequence conservation (B).
